# Parent reports of adolescents and young adults perceived to show signs of a rapid onset of gender dysphoria

**DOI:** 10.1371/journal.pone.0202330

**Published:** 2018-08-16

**Authors:** Lisa Littman

**Affiliations:** Department of Behavioral and Social Sciences, Brown University School of Public Health, Providence, Rhode Island, United States of America; University of Pennsylvania, UNITED STATES

## Abstract

**Purpose:**

In on-line forums, parents have reported that their children seemed to experience a sudden or rapid onset of gender dysphoria, appearing for the first time during puberty or even after its completion. Parents describe that the onset of gender dysphoria seemed to occur in the context of belonging to a peer group where one, multiple, or even all of the friends have become gender dysphoric and transgender-identified during the same timeframe. Parents also report that their children exhibited an increase in social media/internet use prior to disclosure of a transgender identity. Recently, clinicians have reported that post-puberty presentations of gender dysphoria in natal females that appear to be rapid in onset is a phenomenon that they are seeing more and more in their clinic. Academics have raised questions about the role of social media in the development of gender dysphoria. The purpose of this study was to collect data about parents’ observations, experiences, and perspectives about their adolescent and young adult (AYA) children showing signs of an apparent sudden or rapid onset of gender dysphoria that began during or after puberty, and develop hypotheses about factors that may contribute to the onset and/or expression of gender dysphoria among this demographic group.

**Methods:**

For this descriptive, exploratory study, recruitment information with a link to a 90-question survey, consisting of multiple-choice, Likert-type and open-ended questions was placed on three websites where parents had reported sudden or rapid onsets of gender dysphoria occurring in their teen or young adult children. The study’s eligibility criteria included parental response that their child had a sudden or rapid onset of gender dysphoria and parental indication that their child’s gender dysphoria began during or after puberty. To maximize the chances of finding cases meeting eligibility criteria, the three websites (4thwavenow, transgender trend, and youthtranscriticalprofessionals) were selected for targeted recruitment. Website moderators and potential participants were encouraged to share the recruitment information and link to the survey with any individuals or communities that they thought might include eligible participants to expand the reach of the project through snowball sampling techniques. Data were collected anonymously via SurveyMonkey. Quantitative findings are presented as frequencies, percentages, ranges, means and/or medians. Open-ended responses from two questions were targeted for qualitative analysis of themes.

**Results:**

There were 256 parent-completed surveys that met study criteria. The AYA children described were predominantly natal female (82.8%) with a mean age of 16.4 years at the time of survey completion and a mean age of 15.2 when they announced a transgender-identification. Per parent report, 41% of the AYAs had expressed a non-heterosexual sexual orientation before identifying as transgender. Many (62.5%) of the AYAs had reportedly been diagnosed with at least one mental health disorder or neurodevelopmental disability prior to the onset of their gender dysphoria (range of the number of pre-existing diagnoses 0–7). In 36.8% of the friendship groups described, parent participants indicated that the majority of the members became transgender-identified. Parents reported subjective declines in their AYAs’ mental health (47.2%) and in parent-child relationships (57.3%) since the AYA “came out” and that AYAs expressed a range of behaviors that included: expressing distrust of non-transgender people (22.7%); stopping spending time with non-transgender friends (25.0%); trying to isolate themselves from their families (49.4%), and only trusting information about gender dysphoria from transgender sources (46.6%). Most (86.7%) of the parents reported that, along with the sudden or rapid onset of gender dysphoria, their child either had an increase in their social media/internet use, belonged to a friend group in which one or multiple friends became transgender-identified during a similar timeframe, or both

**Conclusion:**

This descriptive, exploratory study of parent reports provides valuable detailed information that allows for the generation of hypotheses about factors that may contribute to the onset and/or expression of gender dysphoria among AYAs. Emerging hypotheses include the possibility of a potential new subcategory of gender dysphoria (referred to as rapid-onset gender dysphoria) that has not yet been clinically validated and the possibility of social influences and maladaptive coping mechanisms. Parent-child conflict may also explain some of the findings. More research that includes data collection from AYAs, parents, clinicians and third party informants is needed to further explore the roles of social influence, maladaptive coping mechanisms, parental approaches, and family dynamics in the development and duration of gender dysphoria in adolescents and young adults.

## Introduction

In recent years, a number of parents have begun reporting in online discussion groups such as 4thwavenow in the US (https://4thwavenow.com) and Transgender Trend in the UK (https://www.transgendertrend.com) that their adolescent and young adult (AYA) children, who have had no histories of childhood gender identity issues, experienced a perceived sudden or rapid onset of gender dysphoria. Parents have described clusters of gender dysphoria in pre-existing friend groups with multiple or even all members of a friend group becoming gender dysphoric and transgender-identified in a pattern that seems statistically unlikely based on previous research [1–8]. Parents describe a process of immersion in social media, such as “binge-watching” YouTube transition videos and excessive use of Tumblr, immediately preceding their child becoming gender dysphoric [1–2, 9]. These types of presentations have not been described in the research literature for gender dysphoria [1–10] and raise the question of whether social influences may be contributing to or even driving these occurrences of gender dysphoria in some populations of adolescents and young adults. (Note: The terminology of “natal sex”, including the terms “natal female” and “natal male”, will be used throughout this article. Natal sex refers to an individual’s sex as it was observed and documented at the time of birth. Some researchers also use the terminology “assigned at birth”.)

## Background

### Gender dysphoria in adolescents

Gender dysphoria (GD) is defined as an individual's persistent discomfort with their biological sex or assigned gender [11]. Two types of gender dysphoria studied include early-onset gender dysphoria, where the symptoms of gender dysphoria begin in early childhood, and late-onset gender dysphoria, where the symptoms begin after puberty [11]. Late-onset gender dysphoria that occurs during adolescence is now called adolescent-onset gender dysphoria. The majority of adolescents who present for care for gender dysphoria are individuals who experienced early-onset gender dysphoria that persisted or worsened with puberty although an atypical presentation has been described where adolescents who did not experience childhood symptoms present with new symptoms in adolescence [7, 12]. Adolescent-onset of gender dysphoria has only recently been reported in the literature for natal females [5,10, 13–14]. In fact, prior to 2012, there were little to no research studies about adolescent females with gender dysphoria first beginning in adolescence [10]. Thus, far more is known about adolescents with early-onset gender dysphoria than adolescents with adolescent-onset gender dysphoria [6, 15]. Although not all research studies on gender dysphoric adolescents exclude those with adolescent-onset gender dysphoria [10], it is important to note that most of the studies on adolescents, particularly those about gender dysphoria persistence and desistance rates and outcomes for the use of puberty suppression, cross-sex hormones, and surgery only included subjects whose gender dysphoria began in childhood and subjects with adolescent-onset gender dysphoria would not have met inclusion criteria for these studies [16–24]. Therefore, most of the research on adolescents with gender dysphoria to date is not generalizable to adolescents experiencing adolescent-onset gender dysphoria [16–24] and the outcomes for individuals with adolescent-onset gender dysphoria, including persistence and desistence rates and outcomes for treatments, are currently unknown.

As recently as 2012, there were only two clinics (one in Canada and one in the Netherlands) that had gathered enough data to provide empirical information about the main issues for gender dysphoric adolescents [25]. Both institutions concluded that the management of adolescent-onset gender dysphoria is more complicated than the management of early-onset gender dysphoria and that individuals with adolescent-onset are more likely to have significant psychopathology [25]. The presentation of gender dysphoria can occur in the context of severe psychiatric disorders, developmental difficulties, or as part of large-scale identity issues and, for these patients, medical transition might not be advisable [13]. The APA Task Force on the Treatment of Gender Identity Disorder notes that adolescents with gender dysphoria “should be screened carefully to detect the emergence of the desire for sex reassignment in the context of trauma as well as for any disorder (such as schizophrenia, mania, psychotic depression) that may produce gender confusion. When present, such psychopathology must be addressed and taken into account prior to assisting the adolescent’s decision as to whether or not to pursue sex reassignment or actually assisting the adolescent with the gender transition.” [25].

### Demographic and clinical changes for gender dysphoria

Although, by 2013, there was research documenting that a significant number of natal males experienced gender dysphoria that began during or after puberty, there was little information about this type of presentation for natal females [5]. Starting in the mid-2000s there has been a substantial change in demographics of patients presenting for care with most notably an increase in adolescent females and an inversion of the sex ratio from one favoring natal males to one favoring natal females [26–28]. And now, some clinicians have noted that they are seeing increasingly in their clinic, the phenomenon of natal females expressing a post-puberty rapid onset of gender dysphoria [14]. Some researchers have suggested that increased visibility of transgender people in the media, availability of information online, with a partial reduction of stigma may explain some of the increases in numbers of patients seeking care [27], but these factors would not explain the reversal of the sex ratio, disproportionate increase in adolescent natal females, and the new phenomenon of natal females experiencing gender dysphoria that begins during or after puberty. If there were cultural changes that made it more acceptable for natal females to seek transition [27], that would not explain why the reversal of the sex ratio reported for adolescents has not been reported for older adult populations [26]. There are many unanswered questions about potential causes for the recent demographic and clinical changes for gender dysphoric individuals.

### Social and peer influences

Parental reports (on social media) of friend clusters exhibiting signs of gender dysphoria [1–4] and increased exposure to social media/internet preceding a child’s announcement of a transgender identity [1–2, 9] raise the possibility of social and peer influences. In developmental psychology research, impacts of peers and other social influences on an individual’s development are sometimes described using the terms peer contagion and social contagion, respectively. The use of “contagion” in this context is distinct from the term’s use in the study of infectious disease, and furthermore its use as an established academic concept throughout this article is not meant in any way to characterize the developmental process, outcome, or behavior as a disease or disease-like state, or to convey any value judgement. Social contagion [29] is the spread of affect or behaviors through a population. Peer contagion, in particular, is the process where an individual and peer mutually influence each other in a way that promotes emotions and behaviors that can potentially have negative effects on their development [30]. Peer contagion has been associated with depressive symptoms, disordered eating, aggression, bullying, and drug use [30–31]. Internalizing symptoms such as depression can be spread via the mechanisms of co-rumination, which entails the repetitive discussion of problems, excessive reassurance seeking (ERS), and negative feedback [30, 32–34]. Deviancy training, which was first described for rule breaking, delinquency, and aggression, is the process whereby attitudes and behaviors associated with problem behaviors are promoted with positive reinforcement by peers [35, 36].

Peer contagion has been shown to be a factor in several aspects of eating disorders. There are examples in the eating disorder and anorexia nervosa literature of how both internalizing symptoms and behaviors have been shared and spread via peer influences [37–41] which may have relevance to considerations of a rapid onset of gender dysphoria occurring in AYAs. Friendship cliques can set the norms for preoccupation with one’s body, one’s body image, and techniques for weight loss, and can predict an individual’s body image concerns and eating behaviors [37–39]. Peer influence is intensified in inpatient and outpatient treatment settings for patients with anorexia and counter-therapeutic subcultures that actively promote the beliefs and behaviors of anorexia nervosa have been observed [39–41]. In these settings, there is a group dynamic where the “best” anorexics (those who are thinnest, most resistant to gaining weight, and who have experienced the most medical complications from their disease) are admired, validated, and seen as authentic while the patients who want to recover from anorexia and cooperate with medical treatment are maligned, ridiculed, and marginalized [39–41]. Additionally, behaviors associated with deceiving parents and doctors about eating and weight loss, referred to as the “anorexic tricks,” are shared by patients in a manner akin to deviancy training [39–41]. Online environments provide ample opportunity for excessive reassurance seeking, co-rumination, positive and negative feedback, and deviancy training from peers who subscribe to unhealthy, self-harming behaviors. The pro-eating disorder sites provide motivation for extreme weight loss (sometimes calling the motivational content “thinspiration”)[42–44]. Such sites promote validation of eating disorder as an identity, and offer “tips and tricks” for weight loss and for deceiving parents and doctors so that individuals may continue their weight-loss activities [42–44]. If similar mechanisms are at work in the context of gender dysphoria, this greatly complicates the evaluation and treatment of impacted AYAs.

**Fig 1:**
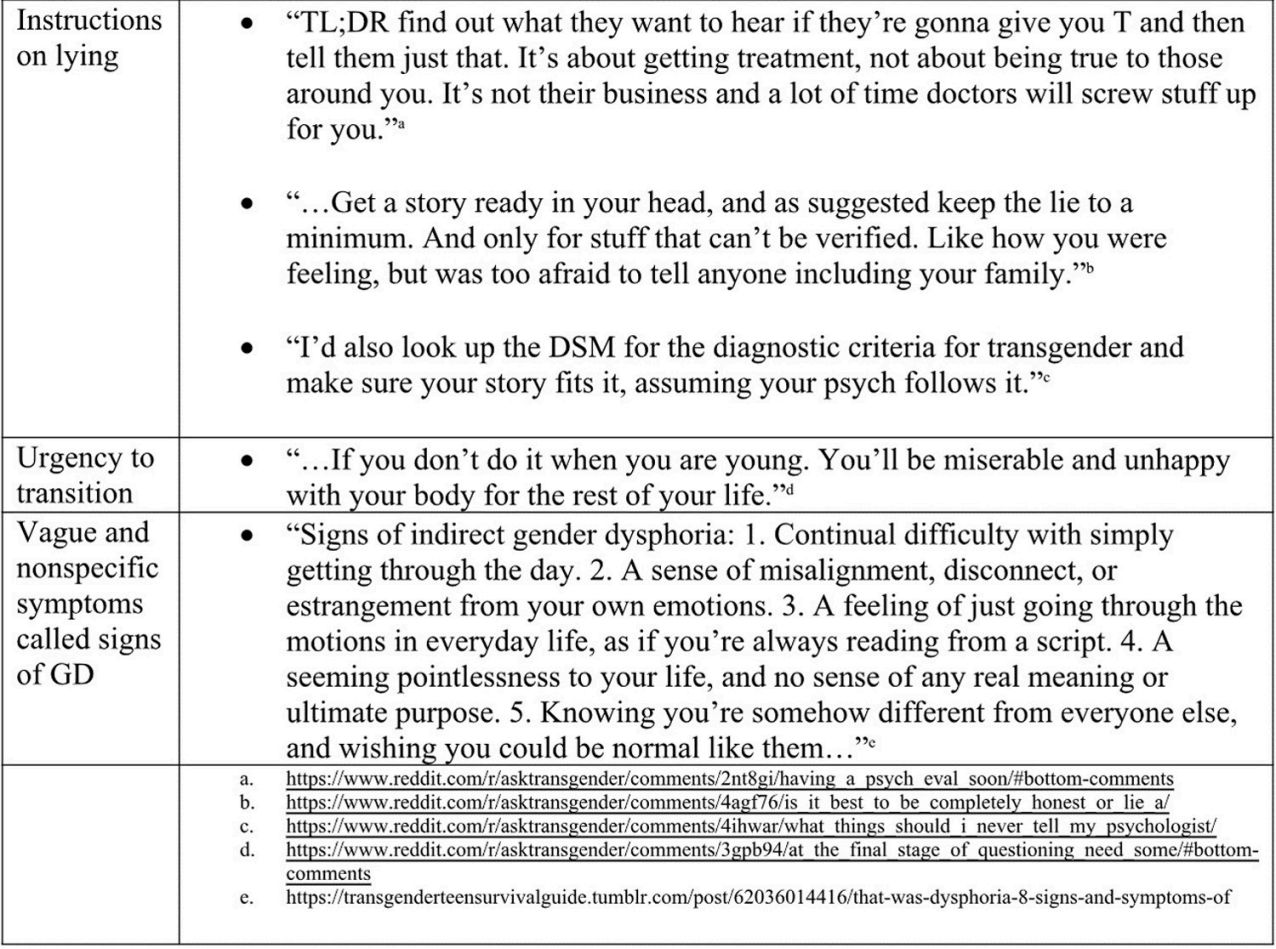
Example quotes of online advice from Reddit and Tumblr.

In the past decade, there has been an increase in visibility, social media, and user-generated online content about transgender issues and transition [45], which may act as a double-edged sword. On the one hand, an increase in visibility has given a voice to individuals who would have been under-diagnosed and undertreated in the past [45]. On the other hand, it is plausible that online content may encourage vulnerable individuals to believe that nonspecific symptoms and vague feelings should be interpreted as gender dysphoria stemming from a transgender condition. Recently, leading international academic and clinical commentators have raised the question about the role of social media and online content in the development of gender dysphoria [46]. Concern has been raised that adolescents may come to believe that transition is the only solution to their individual situations, that exposure to internet content that is uncritically positive about transition may intensify these beliefs, and that those teens may pressure doctors for immediate medical treatment [25]. There are many examples on popular sites such as Reddit (www.reddit.com with subreddit ask/r/transgender) and Tumblr (www.tumblr.com) where online advice promotes the idea that nonspecific symptoms should be considered to be gender dysphoria, conveys an urgency to transition, and instructs individuals how to deceive parents, doctors, and therapists to obtain hormones quickly [47]. Fig 1 includes examples of online advice from Reddit and Tumblr.

### Purpose

Rapid presentations of adolescent-onset gender dysphoria occurring in clusters of pre-existing friend groups are not consistent with current knowledge about gender dysphoria and have not been described in the scientific literature to date [1–8]. The purpose of this descriptive, exploratory research is to (1) collect data about parents’ observations, experiences, and perspectives about their AYA children showing signs of a rapid onset of gender dysphoria that began during or after puberty, and (2) develop hypotheses about factors that may contribute to the onset and/or expression of gender dysphoria among this demographic group.

## Materials and methods

The Icahn School of Medicine at Mount Sinai, Program for the Protection of Human Subjects provided approval of research for this project (HS#: 16–00744).

### Participants

During the recruitment period, 256 parents completed online surveys that met the study criteria. The sample of parents included more women (91.7%) than men (8.3%) and participants were predominantly between the ages of 45 and 60 (66.1%) (Table 1). Most respondents were White (91.4%), non-Hispanic (99.2%), and lived in the United States (71.7%). Most respondents had a Bachelor’s degree (37.8%) or graduate degree (33.1%). The adolescents and young adults (AYAs) described by their parents were predominantly female sex at birth (82.8%) with an average current age of 16.4 years (range, 11–27 years). See Table 2.

**Table 1:** Demographic and other baseline characteristics of parent respondents.

Characteristics of Parent-respondents		n	%
Sex		254	
	Female	233	91.7
	Male	21	8.3
Age (y)		254	
	18–29	3	1.2
	30–44	74	29.1
	45–60	168	66.1
	>60	9	3.5
Race/Ethnicity*		255	
	White	233	91.4
	Other**	22	8.6
Country of Residence		254	
	US	182	71.7
	UK	39	15.4
	Canada	17	6.7
	Other	16	6.3
Education		254	
	Bachelor’s degree	96	37.8
	Graduate degree	84	33.1
	Some college or Associates degree	63	24.8
	HS grad or GED	10	3.9
	<High School	1	0.4
Parent attitude on allowing gay and lesbian couples to marry legally		256	
	Favor	220	85.9
	Oppose	19	7.4
	Don’t know	17	6.6
Parent belief that transgender people deserve the same rights and protections as others		255	
	Yes	225	88.2
	No	8	3.1
	Don’t know	20	7.8
	Other	2	0.8

* may select more than one answer.

** declining order includes: Other, Multiracial, Asian, Hispanic.

**Table 2:** Demographic and other baseline characteristics of AYAs.

Characteristics of AYAs		n	%
AYA sex at birth (natal sex)		256	
	Female	212	82.8
	Male	44	17.2
AYA average current age (range of ages)	16.4 (11–27)	256	
Academic diagnoses		253	
	Gifted	120	47.4
	Learning Disability	11	4.3
	Both	27	10.7
	Neither	95	37.5
Natal female expressed sexual orientation before announcement*		212	
	Asexual	18	8.5
	Bisexual or Pansexual	78	36.8
	Gay or Lesbian	58	27.4
	Straight (Heterosexual)	75	35.4
	Did not express	57	26.9
Natal male expressed sexual orientation before announcement*		44	
	Asexual	4	9.1
	Bisexual or Pansexual	5	11.4
	Gay	5	11.4
	Straight (Heterosexual)	25	56.8
	Did not express	11	25.0
Gender dysphoria began		256	
	During puberty	125	48.8
	After puberty	131	51.2
Along with a rapid onset of GD, the AYA also:		256	
	Belonged to a friend group where one or multiple friends became transgender-identified during a similar timeframe	55	21.5
	Had an increase in social media/internet use	51	19.9
	Both of the above	116	45.3
	Neither	13	5.1
	Don’t know	21	8.2

* may select more than one answer.

### Procedure

A 90-question survey instrument with multiple choice, Likert-type, and open-ended questions was created by the researcher. The survey was designed for parents (respondents) to complete about their adolescent and young adult children. The survey was uploaded onto Survey Monkey (SurveyMonkey, Palo Alto, CA, USA) via an account that was HIPPA-enabled. IRB approval for the study from the Icahn School of Medicine at Mount Sinai in New York, NY was received. Recruitment information with a link to the survey was placed on three websites where parents and professionals had been observed to describe what seemed to be a sudden or rapid onset of gender dysphoria (4thwavenow, transgender trend, and youthtranscriticalprofessionals), although the specific terminology “rapid onset gender dysphoria” did not appear on these websites until the recruitment information using that term was first posted on the sites. Website moderators and potential participants were encouraged to share the recruitment information and link to the survey with any individuals or communities that they thought might include eligible participants to expand the reach of the project through snowball sampling techniques. The survey was active from June 29, 2016 to October 12, 2016 (3.5 months) and took 30–60 minutes to complete. Participants completed the survey at a time and place of their own choosing. Data were collected anonymously and stored securely with Survey Monkey.

Participation in this study was voluntary and its purpose was clearly described in the recruitment information. Electronic consent was obtained. Participants had the option to withdraw consent at any time prior to submitting responses. Inclusion criteria were (1) completion of a survey with parental response that the child had a sudden or rapid onset of gender dysphoria; and (2) parental indication that the child’s gender dysphoria began during or after puberty. There was logic embedded in the survey that disqualified surveys that answered “no” (or skipped the question) about whether the child had a sudden or rapid onset of gender dysphoria and 23 surveys were disqualified prior to completion (20 “no” answers and 3 skipped answers). After cleaning the data for the 274 completed surveys, 8 surveys were excluded for not having a sudden or rapid onset of gender dysphoria and 10 surveys were excluded for not having gender dysphoria that began during or after puberty, which left 256 completed surveys for inclusion. As the survey was voluntary there was no refusal or dropout rate.

### Recruitment sites

There were four sites known to post recruitment information about the research study. The first three were posted due to direct communication with the moderators of the sites. The fourth site posted recruitment information secondary to the snowball sampling technique. The following descriptions provide details about these sites.

### 4thwavenow

4thwavenow was created in 2015. The site, as seen in digitally archived screenshots from 2015 and 2016, stated that it is a “safe place for gender-skeptical parents and their allies”, offered support for parents, and expressed concern about the rush to diagnose young people as transgender and the rush to proceed to medical treatment for them [2, 48]. By June 2016, the site had expanded to include the writing of several parents, “formerly trans-identified people, and people with professional expertise and experience with young people questioning their gender identity” [9]. The perspective of this site might be described as cautious about medical and surgical transition overall—specifically with a cautious or negative view of medical and surgical interventions for children, adolescents, and young adults and an accepting view that mature adults can make their own decisions about transition [2, 9].

### Transgendertrend

Transgendertrend was founded in November 2015. The digitally archived screenshots from November 2015 and July 2016 “Who Are We?” section include the following description, “We are an international group of parents based mainly in the UK, US and Canada, who are concerned about the current trend to diagnose ‘gender non-conforming’ children as transgender. We reject current conservative, reactionary, religious-fundamentalist views about sexuality. We come from diverse backgrounds, some with expertise in child development and psychology, some who were themselves extreme gender non-conforming children and adolescents, some whose own children have self-diagnosed as ‘trans’ and some who know supportive trans adults who are also questioning recent theories of ‘transgenderism’” [49]. In July of 2016, there was additional text added, expressing concern about legislation regarding public bathrooms and changing rooms [50].

### Youth trans critical professionals

Youth Trans Critical Professionals was created in March 2016. The digitally archived screenshot from the April 2016 “About” section stated the following: “This website is a community of professionals “thinking critically about the youth transgender movement. We are psychologists, social workers, doctors, medical ethicists, and academics. We tend to be left-leaning, open-minded, and pro-gay rights. However, we are concerned about the current trend to quickly diagnose and affirm young people as transgender, often setting them down a path toward medical transition. Our concern is with medical transition for children and youth. We feel that unnecessary surgeries and/or hormonal treatments which have not been proven safe in the long-term represent significant risks for young people” [51].

### Parents of transgender children

Parents of Transgender Children is a private Facebook group with more than 8,000 members [52]. The current “About” section states that requests to join the group “will be denied if you are not the parent (or immediate caregiver or family member) of a transgender, gender-fluid, gender-questioning, agender, or other gender-nonconforming child (of any age); or if you are uncooperative during screening” and that the “group is comprised of parents and parenting figures, as well as a select group of advocates INVITED by the admin[istrative] staff to assist & help us with understanding legal and other concerns” [52]. Although the parent discussions and comments are not viewable to non-members [52], this group is perceived to be pro -gender-affirming. The Parents of Transgender Children Facebook group is considered to be a site to find parents who are supportive of their child’s gender identity [53], and it is listed as a resource in a gender affirming parenting guide [54] and by gender affirming organizations [55–56].

## Measures

### Basic demographic and baseline characteristics

Basic demographic and baseline characteristic questions, including parental attitudes about LGBT rights, were included. Parents were asked about their children’s mental health disorders and neurodevelopmental disabilities that were diagnosed before their child’s onset of gender dysphoria as well as during and after. The question, “Has your child been formally identified as academically gifted, learning disabled, both, neither?” was used as a proxy to estimate rates of academic giftedness and learning disabilities. Questions about trauma and non-suicidal self-injury were also included as were questions about social difficulties described in a previous research study about gender dysphoric adolescents [13].

### DSM-5 diagnostic criteria for gender dysphoria in children

The DSM 5 criteria for gender dysphoria in children consist of eight indicators of gender dysphoria [57]. To meet criteria for diagnosis, a child must manifest at least six out of eight indicators including the one designated A1, “A strong desire to be the other gender or an insistence that one is the other gender (or some alternative gender different from one’s assigned gender).” Three of the indicators (A1, A7, and A8) refer to desires or dislikes of the child. Five of the indicators (A2-A6) are readily observable behaviors and preferences such as a strong preference or strong resistance to wearing certain kinds of clothing; a strong preference or strong rejection of specific toys, games and activities; and a strong preference for playmates of the other gender [57]. The eight indicators were simplified for language and parents were asked to note which, if any, their child had exhibited prior to puberty. The requirement of six-month duration of symptoms was not included.

### DSM-5 diagnostic criteria for gender dysphoria in adolescents and adults

The DSM-5 criteria for gender dysphoria in adolescents and adults consist of six indicators of gender dysphoria [57]. To meet criteria for diagnosis, an adolescent or adult must manifest at least two of the six indicators. The six indicators were simplified for language, the first indicator was adjusted for a parent to answer about their child, and parents were asked to note which, if any, their child was expressing currently. The requirement of six-month duration of symptoms was not included.

### Exposure to friend groups and social media/internet content

Survey questions were developed to describe AYA friend groups, including number of friends that became transgender-identified in a similar time period as the AYA, peer group dynamics and behaviors, and exposure to specific types of social media/internet content and messages that have been observed on sites popular with teens, such as Reddit and Tumblr.

### Behaviors, outcomes, clinical interactions

Survey questions were developed to specifically quantify adolescent behaviors that had been described by parents in online discussions and observed elsewhere. Participants were asked to describe outcomes such as their child’s mental well-being and parent-child relationship since becoming transgender-identified. Parents were also asked about experiences with clinicians and their children’s disposition regarding steps taken for transition and duration of transgender-identification both for children who were still transgender-identified and for children who were no longer transgender-identified.

### Coping with strong or negative emotions

Two questions about the AYAs’ ability to cope with negative and strong emotions were included. One question was “How does your child handle strong emotions? (please select the best answer).” Offered answers were “My child is overwhelmed by strong emotions and goes to great lengths to avoid feeling them,” “My child is overwhelmed by strong emotions and tries to avoid feeling them,” “My child neither avoids not seeks out strong emotions,” “My child tries to seek out situations in order to feel strong emotions,” “My child goes to great lengths to seek out situations in order to feel strong emotions,” “None of the above,” “I don’t know.” The other question was “How would you rate your child’s ability to deal with their negative emotions and channel them into something productive?” An example was given regarding dealing with a low test grade by studying harder for the next test (excellent) or by ignoring it, throwing a tantrum, blaming the teacher or distracting themselves with computer games, alcohol, drugs, etc. (extremely poor). Offered answers were: excellent, good, fair, poor, extremely poor, and I don’t know.

## Data analysis

Statistical analyses of quantitative data were performed using Excel and custom shell scripts (Unix). Quantitative findings are presented as frequencies, percentages, ranges, means and/or medians. ANOVAs, chi-squared, and t-tests comparisons were used where appropriate using publicly available calculators and p<0.05 was considered significant. Qualitative data were obtained from open text answers to questions that allowed participants to provide additional information or comments. The types of comments and descriptions were categorized, tallied, and reported numerically. A grounded theory approach was selected as the analytic strategy of choice for handling the qualitative responses because it allowed the researcher to assemble the data in accordance with the salient points the respondents were making without forcing the data into a preconceived theoretical framework of the researcher’s own choosing [58]. Illustrative respondent quotes and summaries from the qualitative data are used to illustrate the quantitative results and to provide relevant examples. Two questions were targeted for full qualitative analysis of themes (one question on friend group behaviors and one on clinician interactions). For these questions, a second reviewer with expertise in qualitative methods was engaged (MM). Both the author (LL) and reviewer (MM) independently analyzed the content of the open text answers and identified major themes. Discrepancies were resolved with collaborative discussion and themes were explored and refined until agreement was reached for the final lists of themes. Representative quotes for each theme were selected by LL, reviewed by MM, and agreement was reached.

## Results

### Baseline characteristics

Baseline characteristics (Table 1) included that the vast majority of parents favored gay and lesbian couples’ right to legally marry (85.9%) and believed that transgender individuals deserve the same rights and protections as other individuals in their country (88.2%). Along with the sudden or rapid onset of gender dysphoria, the AYAs belonged to a friend group where one or multiple friends became gender dysphoric and came out as transgender during a similar time as they did (21.5%), exhibited an increase in their social media/internet use (19.9%), both (45.3%), neither (5.1%), and don’t know (8.2%) (Table 2). For comparisons, the first three categories will be combined and called “social influence” (86.7%) and the last two combined as “no social influence” (13.3%). Nearly half (47.4%) of the AYAs had been formally diagnosed as academically gifted, 4.3% had a learning disability, 10.7% were both gifted and learning disabled, and 37.5% were neither. Sexual orientation as expressed by the AYA prior to transgender-identification is listed separately for natal females and for natal males (Table 2). Overall, 41% of the AYAs expressed a non-heterosexual sexual orientation prior to disclosing a transgender-identification.

It is important to note that none of the AYAs described in this study would have met diagnostic criteria for gender dysphoria in childhood (Table 3). In fact, the vast majority (80.4%) had zero indicators from the DSM-5 diagnostic criteria for childhood gender dysphoria with 12.2% possessing one indicator, 3.5% with two indicators, and 2.4% with three indicators. Breaking down these results, for readily observable indicators (A2-6), 83.5% of AYAs had zero indicators, 10.2% had one indicator, 3.9% had two indicators, and 1.2% had three indicators. For the desire/dislike indicators (A1, A7, A8), which a parent would have knowledge of if the child expressed them verbally, but might be unaware if a child did not, 95.7% had zero indicators and 3.5% had one indicator. Parents responded to the question about which, if any, of the indicators of the DSM criteria for adolescent and adult gender dysphoria their child was experiencing currently. The average number of positive current indicators was 3.5 (range 0–6) and 83.2% of the AYA sample was currently experiencing two or more indicators. Thus, while the focal AYAs did not experience childhood gender dysphoria, the majority of those who were the focus of this study were indeed gender dysphoric at the time of the survey completion.

**Table 3:** DSM 5 Indicators for gender dysphoria.

Characteristics		n	%
AYAs who would have met diagnostic criteria for gender dysphoria in childhood		0	0
Number of DSM 5 indicators for gender dysphoria in children exhibited prior to puberty		255	
	Zero indicators	205	80.4
	One indicator	31	12.2
	Two indicators	9	3.5
	Three indicators	6	2.4
	Four indicators	3	1.2
Desire/Dislike Indicators (A1, A7, or A8)		255	
	Zero indicators	244	95.7
	One indicators	9	3.5
	Two indicators	0	0
	Three indicators	1	0.4
Readily observable indicators (A2-A6)		254	
	Zero indicators	212	83.5
	One indicator	26	10.2
	Two indicators	10	3.9
	Three indicators	3	1.2
	Four indicators	3	1.2
Average number of DSM 5 indicators for adolescent and adult gender dysphoria that the AYA is experiencing currently (range)			
	3.5 (range 0–6)	247	
AYAs currently experiencing two or more indicators of gender dysphoria for adolescents and adults		250	
	Yes	208	83.2
	No	40	16.0
	Don’t know	2	0.8

The AYAs who were the focus of this study had many comorbidities and vulnerabilities predating the onset of their gender dysphoria, including psychiatric disorders, neurodevelopmental disabilities, trauma, non-suicidal self-injury (NSSI), and difficulties coping with strong or negative emotions (Table 4). The majority (62.5%) of AYAs had one or more diagnoses of a psychiatric disorder or neurodevelopmental disability preceding the onset of gender dysphoria (range of the number of pre-existing diagnoses 0–7). Many (48.4%) had experienced a traumatic or stressful event prior to the onset of their gender dysphoria. Open text descriptions of trauma were categorized as “family” (including parental divorce, death of a parent, mental disorder in a sibling or parent), “sex or gender related” (such as rape, attempted rape, sexual harassment, abusive dating relationship, break-up), “social” (such as bullying, social isolation), “moving” (family relocation or change of schools); “psychiatric” (such as psychiatric hospitalization), and medical (such as serious illness or medical hospitalization). Almost half (45.0%) of AYAs were engaging in non-suicidal self-injury (NSSI) behavior before the onset of gender dysphoria. Coping styles for these AYAs included having a poor or extremely poor ability to handle negative emotions productively (58.0%) and being overwhelmed by strong emotions and trying to avoid (or go to great lengths to avoid) experiencing them (61.4%) (Table 4). The majority of respondents (69.4%) answered that their child had social anxiety during adolescence; 44.3% that their child had difficulty interacting with their peers, and 43.1% that their child had a history of being isolated (not associating with their peers outside of school activities).

**Table 4:** AYA baseline comorbidities and vulnerabilities predating the onset of gender dysphoria.

Characteristics		n	%
Mental disorder or neurodevelopmental disability diagnosed prior to the onset of gender dysphoria*		251	
	Anxiety	117	46.6
	Depression	99	39.4
	Attention Deficit Hyperactivity Disorder (ADHD)	29	11.6
	Obsessive Compulsive Disorder (OCD)	21	8.4
	Autism Spectrum Disorder (ASD)	20	8.0
	Eating Disorder	12	4.8
	Bipolar Disorder	8	3.2
	Psychosis	6	2.4
	None of above	94	37.5
	(Other) Borderline	3	1.2
	(Other) Oppositional Defiant Disorder	2	0.8
Traumatic or stressful experience prior to the onset of gender dysphoria		252	
	Yes	122	48.4
	No	91	36.1
	Don't know	38	15.1
	Other	1	0.4
Types of trauma*		113	
	Family	50	44.2
	Sex/Gender related	34	30.1
	Social	23	20.4
	Moving	20	17.7
	Psychiatric	9	8.0
	Medical	7	6.2
Non-suicidal self-injury (NSSI) before the onset of gender dysphoria		180	
		81	45.0
Ability to handle negative emotions productively		255	
	Excellent/Good	34	13.3
	Fair	70	27.5
	Poor/Extremely Poor	148	58.0
	Don't know	3	1.2
Coping style for dealing with strong emotions		254	
	Overwhelmed by strong emotions and tries to /goes to great lengths to avoid feeling them	156	61.4
	Neither avoids nor seeks out strong emotions	29	11.4
	Tries to/goes to great lengths to seeks out strong emotions	33	13.0
	Don’t know	25	9.8
	None of the above	11	4.3
Social vulnerabilities		255	
	During adolescence child had social anxiety	177	69.4
	Child had difficulty interacting with their peers	113	44.3
	History of being isolated (not interacting with peers outside of school activities)	110	43.1
	Child felt excluded by peers throughout most of grade school	93	36.5
	Child had persistent experiences of being bullied before the onset of gender dysphoria	74	29.0

*may select more than one answer.

### Announcing a transgender-identification

At the time the AYA announced they were transgender-identified (“came out”), most were living at home with one or both parents (88.3%) and a small number were living at college (6.2%). The average age of announcement of a transgender-identification was 15.2 years of age (range 10–21) (Table 5). Most of the parents (80.9%) answered affirmatively that their child’s announcement of being transgender came “out of the blue without significant prior evidence of gender dysphoria.” Respondents were asked to pinpoint a time when their child seemed not at all gender dysphoric and to estimate the length of time between that point and their child’s announcement of a transgender-identity. Almost a third of respondents (32.4%) noted that their child did not seem gender dysphoric when they made their announcement and 26.0% said the length of time from not seeming gender dysphoric to announcing a transgender identity was between less than a week to three months. The most striking examples of “not seeming at all gender dysphoric” prior to making the announcement included a daughter who loved summers and seemed to love how she looked in a bikini, another daughter who happily wore bikinis and makeup, and another daughter who previously said, “I love my body!”

The majority of respondents (69.2%) believed that their child was using language that they found online when they “came out.” A total of 130 participants provided optional open text responses to this question, and responses fell into the following categories: why they thought the child was using language they found online (51); description of what the child said but didn’t provide a reason that they suspected the child was using language they found online (61); something else about the conversation (8) or the child (7) and don’t know (3). Of the 51 responses describing reasons why respondents thought their child was reproducing language they found online, the top two reasons were that it didn’t sound like their child’s voice (19 respondents) and that the parent later looked online and recognized the same words and phrases that their child used when they announced a transgender identity (14 respondents). The observation that it didn’t sound like their child’s voice was also expressed as “sounding scripted,” like their child was “reading from a script,” “wooden,” “like a form letter,” and that it didn’t sound like their child’s words. Parents described finding the words their child said to them “verbatim,” “word for word,” “practically copy and paste,” and “identical” in online and other sources. The following quotes capture these top two observations. One parent said, “It seemed different from the way she usually talked—I remember thinking it was like hearing someone who had memorized a lot of definitions for a vocabulary test.” Another respondent said, “The email [my child sent to me] read like all of the narratives posted online almost word for word.”

**Table 5:** Announcing a transgender-identification.

Characteristics		n	%
Age of AYA when the AYA announced a transgender-identification (range)	15.2 average (10–21)	255	
Living arrangement at announcement		256	
	Living at home with one or both parents	226	88.3
	Living at college or university	16	6.2
	Other	14	5.5
AYA’s announcement came from “out of the blue, without significant prior evidence of gender dysphoria”		256	
	Yes	207	80.9
	No	33	12.9
	Other	16	6.2
If a time was pinpointed when the child seemed not at all gender dysphoric, how long between that time and the child’s announcement of a transgender-identity?		250	
	Did not seem at all gender dysphoric when they announced and transgender-identity	81	32.4
	Less than a week to 3 months	65	26.0
	4–6 months	31	12.4
	7–9 months	10	4.0
	10–12 months	29	11.6
	More than 12 months	20	8.0
	Don’t know	14	5.6
Parent suspects that when the child first announced a transgender-identity, that the child used language that they found online		253	
	Yes	175	69.2
	No	53	20.9
	N/A	25	9.9
Parent thinks their child is correct in their child’s belief of being transgender		255	
	Yes	6	2.4
	No	195	76.5
	Don’t know	38	14.9
	Other	16	6.3
How soon after the announcement did the AYA ask for transition?		255	
	At the same time	86	33.7
	Between less than one week to one month	33	12.9
	2–5 months after announcement	26	10.2
	6 or more months after announcement	19	7.5
	Other	16	6.3
	N/A	75	29.4
Intention and request for transition*		189	
	AYA told the parent that they want cross-sex hormones	127	67.2
	AYA told the parent that they want to go to a gender therapist/gender clinic	111	58.7
	AYA told the parent that they want surgery	101	53.4
	AYA brought up the issue of suicides in transgender teens as a reason that their parent should agree to treatment	59	31.2
AYA has very high expectation that transitioning will solve their problems in social, academic, occupational, or mental health areas		256	
	Yes	143	55.9
	No	13	5.1
	Don’t know	100	39.1
AYA was willing to work on basic mental health before seeking gender treatments		253	
	Yes	111	43.9
	No	71	28.1
	Don’t know	30	11.9
	N/A	41	16.2

*may select more than one answer.

The following case summaries were selected to illustrate peer, trauma, and psychiatric contexts that might indicate more complicated clinical pictures.

- A 12-year-old natal female was bullied specifically for going through early puberty and the responding parent wrote “as a result she said she felt fat and hated her breasts.” She learned online that hating your breasts is a sign of being transgender. She edited her diary (by crossing out existing text and writing in new text) to make it appear that she has always felt that she is transgender.
- A 14-year-old natal female and three of her natal female friends were taking group lessons together with a very popular coach. The coach came out as transgender, and, within one year, all four students announced they were also transgender.
- A natal female was traumatized by a rape when she was 16 years of age. Before the rape, she was described as a happy girl; after the rape, she became withdrawn and fearful. Several months after the rape, she announced that she was transgender and told her parents that she needed to transition.
- A 21-year-old natal male who had been academically successful at a prestigious university seemed depressed for about six months. Since concluding that he was transgender, he went on to have a marked decline in his social functioning and has become increasingly angry and hostile to his family. He refuses to move out or look for a job. His entire family, including several members who are very supportive of the transgender community, believe that he is “suffering from a mental disorder which has nothing to do with gender.”
- A 14-year-old natal female and three of her natal female friends are part of a larger friend group that spends much of their time talking about gender and sexuality. The three natal female friends all announced they were trans boys and chose similar masculine names. After spending time with these three friends, the 14-year-old natal female announced that she was also a trans boy.

The majority (76.5%) of the surveyed parents felt that their child was incorrect in their belief of being transgender (Table 5). More than a third (33.7%) of the AYAs asked for medical and/or surgical transition at the same time that they announced they were transgender-identified. Two thirds (67.2%) of the AYAs told their parent that they wanted to take cross-sex hormones; 58.7% that they wanted to see a gender therapist/gender clinic; and 53.4% that they wanted surgery for transition. Almost a third (31.2%) of AYAs brought up the issue of suicides in transgender teens as a reason that their parent should agree to treatment. More than half of the AYAs (55.9%) had very high expectations that transitioning would solve their problems in social, academic, occupational or mental health areas. While 43.9% of AYAs were willing to work on basic mental health before seeking gender treatments, a sizable minority (28.1%) were not willing to work on their basic mental health before seeking gender treatment. At least two parents relayed that their child discontinued psychiatric care and medications for pre-existing mental health conditions once they identified as transgender. One parent, in response to the question about if their child had very high expectations that transitioning would solve their problems elaborated, “Very much so. [She] discontinued anti-depressant quickly, stopped seeing psychiatrist, began seeing gender therapist, stopped healthy eating. [She] stated ‘none of it’ (minding what she ate and taking her Rx) ‘mattered anymore.’ This was her cure, in her opinion.”

### Friend-group exposure

The adolescent and young adult children were, on average, 14.4 years old when their first friend became transgender-identified (Table 6). Within friendship groups, the average number of individuals who became transgender-identified was 3.5 per group. In 36.8% of the friend groups described, the majority of individuals in the group became transgender-identified. The order that the focal AYA “came out” compared to the rest of their friendship group was calculated from the 119 participants who provided the number of friends coming out both before and after their child and 74.8% of the AYAs were first, second or third of their group. Parents described intense group dynamics where friend groups praised and supported people who were transgender-identified and ridiculed and maligned non-transgender people. Where popularity status and activities were known, 60.7% of the AYAs experienced an increased popularity within their friend group when they announced a transgender-identification and 60.0% of the friend groups were known to mock people who were not transgender or LGBTIA (lesbian, gay, bisexual, transgender, intersex, or asexual).

**Table 6:** Friend group exposure.

Characteristics		n	%
The AYA has been part of a friend group where one or more friends has come out as transgender around a similar timeframe as they did		254	
	Yes	176	69.3
	No	47	18.5
	Don’t know	31	12.2
Age of AYA when their first friend became transgender-identified (range)	14.4 average (11–21)	174	
Number of friends from the friendship group who became gender dysphoric average (range)	3.5 average (2–10)	138	
Where numbers known, friend groups where the MAJORITY of the friends in the friendship group became transgender-identified		125	
	Yes	46	36.8
	No	79	63.2
Order of the AYAs “coming out” compared to the others in the friendship group		119	
	First in the friendship group	4	3.4
	Second in the friendship group	52	43.7
	Third in the friendship group	33	27.7
	Fourth in the friendship group	18	15.1
	Fifth in the friendship group	5	4.2
	Sixth or Seventh in the friendship group	6	5.0
Where popularity status known, change in popularity within friend group when AYA announced their transgender-identification		178	
	Increased popularity	108	60.7
	Decreased popularity	11	6.2
	Unchanged popularity	59	33.1
Where friend group activities known, friend group known to mock people who are not transgender/LGBT		145	
	Yes	87	60.0
	No	58	40.0

For the question about popularity changes when the child came out as having a transgender-identification, 79 participants provided optional open text responses which were categorized as: descriptions of the responses the child received (39); descriptions of the friends (14); description that the child did not “come out” to friends (8); not sure (9); speculation on how the child felt from the response (4), other (5). Of the 39 descriptions of responses, 19 of these responses referred to positive benefits the child received after coming out including positive attention, compliments, increased status, increased popularity, increased numbers of online followers, and improved protection from ongoing bullying. The following are quotes from parents about the perceived benefits of transgender-identification afforded to their child. One respondent said, “Great increase in popularity among the student body at large. Being trans is a gold star in the eyes of other teens.” Another respondent explained, “not so much ‘popularity’ increasing as ‘status’…also she became untouchable in terms of bullying in school as teachers who ignored homophobic bullying …are now all at pains to be hot on the heels of any trans bullying.” Seven respondents described a mixed response where the child’s popularity increased with some friends and decreased with others. Seven respondents described a neutral response such as “All of the friends seemed extremely accepting.” Two described a temporary increase in their child’s popularity: “There was an immediate rush of support when he came out. Those same friends have dwindled to nothing as he rarely speaks to any of them now.” Another described the loss of friends. And two parents described that “coming out” prevented the loss of friends explained by one respondent as “to not be trans one would not have been included in his group.”

Several AYAs expressed significant concern about the potential repercussions from their friend group when they concluded that they were not transgender after all. There were two unrelated cases with similar trajectories where the AYAs spent some significant time in a different setting, away from their usual friend group, without access to the internet. Parents described that these AYAs made new friendships, became romantically involved with another person, and during their time away concluded that they were not transgender. In both cases, the adolescents, rather than face their school friends, asked to move and transfer to different high schools. One parent said that their child, “…couldn’t face the stigma of going back to school and being branded as a fake or phony. … Or worse, a traitor or some kind of betrayer…[and] asked us if we could move.” In the other case, the parent relayed that their child thought none of the original friends would understand and expressed a strong desire to “…get out of the culture that ‘if you are cis, then you are bad or oppressive or clueless.’” Both families were able to relocate and both respondents reported that their teens have thrived in their new environments and new schools. One respondent described that their child expressed relief that medical transition was never started and felt there would have been pressure to move forward had the family not moved away from the peer group.

### Qualitative analysis

The open-ended responses from the question about whether the AYAs and friends mocked, teased, or made fun of individuals who weren’t transgender or LGBTIA was selected for additional qualitative analysis. Seven major themes were identified from the comments provided by participants and are described, with representative supporting quotes.

#### Theme: Groups targeted

The groups targeted for mocking by the friend groups are often heterosexual (straight) people and non-transgender people (called “cis” or “cisgender”). Sometimes animosity was also directed towards males, white people, gay and lesbian (non-transgender) people, aromantic and asexual people, and “terfs”. One participant explained, “They are constantly putting down straight, white people for being privileged, dumb and boring.” Another participant elaborated, “In general, cis-gendered people are considered evil and unsupportive, regardless of their actual views on the topic. To be heterosexual, comfortable with the gender you were assigned at birth, and non-minority places you in the ‘most evil’ of categories with this group of friends. Statement of opinions by the evil cis-gendered population are consider phobic and discriminatory and are generally discounted as unenlightened.”

#### Theme: Individuals targeted

In addition to targeting specific groups of people for mocking, the AYAs and their friend groups also directed mocking towards individuals in the AYAs’ lives such as parents, grandparents, siblings, peers, allies, and teachers. The following quotes describe individuals targeted. One participant said, “They call kids who are not LGBT dumb and cis. And the mocking has been aimed at my transgender-identified child’s [sibling].” Another parent said, “They definitely made fun of parents and teachers who did not agree with them.” And a third participant said, “…they were asked to leave [a school-based LGBT club] because they were not queer enough [as straight and bisexual allies]. [One of them] was [then] bullied, harassed and denounced online.”

#### Theme: Behaviors occurred both in person and in online settings

Parents observed the behaviors both in-person and in online settings, and specifically mentioned seeing posts and conversations on Tumblr, Twitter, Facebook, and Instagram. On participant said, “They speak with derision about how cis-gendered people do not understand them and are so close-minded.” Another participant said, “I hear them disparaging heterosexuality, marriage and nuclear families.” Another participant said, “On my daughter's Tumblr blog, she has liked or favorited or re-posted disparaging comments about those who aren't transgender or seem to misunderstand the transgender identity.” And another parent reported, “Her real life friends don't [mock non-LGBT people] but online they are always swapping jokes and comments about cisgender and about transphobia.”

#### Theme: Examples of behaviors

Participants gave many examples of the observed behaviors that were mocking towards non-transgender people and non-LGB people. One participant said, “My daughter called me a ‘breeder’ and says things in a mocking ‘straight person voice’. Her friends egg her on when she does this.” Another parent offered, “If they aren't mocking ‘cis’ people, they are playing pronoun police and mocking people who can't get the pronouns correct.” Another participant said, “New vocabulary includes ‘cis-stupid’ and ‘cis-stupidity.’” And a fourth participant described, “They assume anyone that is critical about being transgender (even just asking questions) is either ignorant or filled with hate.”

#### Theme: Emphasizing victimhood

Participants described that their children and friend group seemed to focus on feeling as though they were victims. One participant described, “They seem to wear any problems they may have, real or perceived like badges of honor…I feel like they want to believe they are oppressed & have really 'been through life', when they have little life experience.” Another participant said, “…there is a lot of feeling like a victim [and being] part of a victimized club.” Another parent said “But all talk is very 'victim' centered”. And finally, another said, “They passionately decry ‘Straight Privilege’ and ‘White Male Privilege’—while emphasizing their own ‘Victimhood.’”

#### Theme: Consequences of behaviors

A few participants describe that because of their child’s behavior, there were consequences, including making it difficult for one child to return to her school and the following description from another parent, “Most relatives have blocked her on [social media] over constant jokes regarding cis and straight people.”

#### Theme: Fueling the behaviors

In some cases, parents describe a synergistic effect of kids encouraging other kids to persist in the behavior as was described in a previous quote, “Her friends egg her on when she does this” as well as the following, “Lots of discussion revolving around how their teachers ‘discriminate’ or are ‘mean’ to them based on their declared LGBTIA identity, and they get each other riled up convincing each other of their persecution by these perceived wrongs … privately they mock our intolerance, and in person act upon these false beliefs by treating us as people out to get them…”

### Internet/social media exposure

In the time period just before announcing that they were transgender, 63.5% of AYAs exhibited an increase in their internet/social media (Table 7). To assess AYA exposure to existing online content, parents were asked what kind of advice their child received from someone/people online. AYAs had received online advice including how to tell if they were transgender (54.2%); the reasons that they should transition right away (34.7%); that if their parents did not agree for them to take hormones that the parents were “abusive” and “transphobic” (34.3%); that if they waited to transition they would regret it (29.1%); what to say and what not to say to a doctor or therapist in order to convince them to provide hormones (22.3%); that if their parents were reluctant to take them for hormones that they should use the “suicide narrative” (telling the parents that there is a high rate of suicide in transgender teens) to convince them (20.7%); and that it is acceptable to lie or withhold information about one’s medical or psychological history from a doctor or therapist in order to get hormones/get hormones faster (17.5%). Two respondents, in answers to other questions, described that their children later told them what they learned from online discussion lists and sites. One parent reported, “He has told us recently that he was on a bunch of discussion lists and learned tips there. Places where teens and other trans people swap info. Like to use [certain, specific] words [with] the therapist when describing your GD, because [they are] code for potentially suicidal and will get you a diagnosis and Rx for hormones.” Another parent disclosed, “The threat of suicide was huge leverage. What do you say to that? It’s hard to have a steady hand and say no to medical transition when the other option is dead kid. She learned things to say that would push our buttons and get what she wanted and she has told us now that she learned that from trans discussion sites.”

**Table 7:** Internet/Social media exposures.

		n	%
AYAs internet/social media use just prior to announcement		255	
	Increased social media/internet use	162	63.5
	Decreased social media/internet use	3	1.2
	Unchanged social media/internet use	49	19.2
	Don’t know	41	16.1
AYA exposure to internet content/advice*		251	
	How to tell if they are transgender	136	54.2
	The reasons that they should transition right away	87	34.7
	That if their parents did not agree to take them for hormones, that the parents are “abusive” and “transphobic”	86	34.3
	That if they waited to transition they would regret it	73	29.1
	That if they didn’t transition immediately they would never be happy	72	28.7
	How to order physical items (binders, packers, etc) without parents finding out	67	26.7
	What to say and what NOT to say to a doctor or therapist in order to convince them to provide hormones	56	22.3
	That if their parents are reluctant to take them for hormones, that they should use the “suicide narrative” to convince them (telling the parents that there is a high rate of suicide in transgender teens.)	52	20.7
	Medical advice about the risks and benefits of hormones	55	21.9
	Medical advice about the risks and benefits of surgery	47	18.7
	That it is acceptable to lie to or withhold information about one’s medical or psychological history from a doctor or therapist in order to get hormones/get hormones faster	44	17.5
	How to hide physical items from parents	40	15.9
	How to hide or make excuses for physical changes	26	10.4
	How to get money from others online in order to pay for medications, etc	25	10.0
	How to get hormones from online sources	24	9.6
	How to hide hormones from parents	21	8.4
	I don’t know if my child received online advice about these topics	127	50.6

*may select more than one answer.

Parents identified the sources they thought were most influential for their child becoming gender dysphoric. The most frequently answered influences were: YouTube transition videos (63.6%); Tumblr (61.7%); a group of friends they know in person (44.5%); a community/group of people that they met online (42.9%); a person they know in-person (not online) 41.7%. In contrast to the majority of responses, two participants commented that they didn’t think the sources influenced their child to become gender dysphoric, rather they gave their child a name for their feelings or gave the child confidence to come out. The following quotes illustrate the dominant quantitative findings. One parent wrote, “We believe the biggest influence was the online pro-transition blogs and youtube videos. We feel she was highly influenced by the ‘if you are even questioning your gender-you are probably transgender’ philosophy…In the ‘real world’ her friends, other trans peers, and newfound popularity were additional areas of reinforcement.” Another respondent described the online influence as part of a different question, “I believe my child experienced what many kids experience on the cusp of puberty—uncomfortableness!—but there was an online world at the ready to tell her that those very normal feelings meant she's in the wrong body.”

### Mental well-being, mental health, and behaviors

The trajectories of the AYAs were not consistent with the narrative of discovering one’s authentic self and then thriving. Specifically, parents reported that, after “coming out,” their children exhibited a worsening of their mental well-being. Additionally, parents noted worsening of the parent-child relationship and observed that their children had narrowed their interests (Table 8). Although small numbers of AYAs had improvement in mental well-being (12.6%), parent-child relationship (7.4%), grades/academic performance (6.4%), and had broadened their interests and hobbies (5.1%); the most common outcomes were worsened mental well-being (47.2%); worsened parent child relationship (57.3%); unchanged or mixed grades/academic performance (59.1%); and a narrowed range of interests and hobbies (58.1%). One parent describing her child’s trajectory offered, “After announcing she was transgender, my daughter’s depression increased significantly. She became more withdrawn. She stopped participating in activities which she previously enjoyed, stopped participating in family activities, and significantly decreased her interaction with friends. Her symptoms became so severe that she was placed on medication by her physician.” Table 9 describes cumulative rates of mental illness and neurodevelopmental disability at the time of survey.

**Table 8:** Outcomes and behaviors.

Characteristics		n	%
AYA mental well-being since announcement		254	
	Worse	120	47.2
	Better	32	12.6
	Unchanged or mixed	101	39.8
	Don’t know	1	0.4
Parent-child relationship since announcement		253	
	Worse	145	57.3
	Better	18	7.4
	Unchanged or mixed	89	35.2
	Don’t know	1	0.4
Grades/academic performance		220	
	Worse	76	34.5
	Better	14	6.4
	Unchanged/mixed	130	59.1
Range of interests and hobbies		255	
	Much broader	2	0.8
	Somewhat broader	11	4.3
	Unchanged	93	36.5
	Somewhat narrower	64	25.1
	Much narrower	56	22.0
	There are very few topics outside of transgender issues that my child is interested in	28	11.0
	Don/t know	1	0.4

**Table 9:** AYA Cumulative mental disorder and neurodevelopmental disability diagnoses.

Characteristics		n	%
Mental disorder or neurodevelopmental disability		243	
	Anxiety	154	63.4
	Depression	143	58.8
	Attention Deficit Hyperactivity Disorder (ADHD)	36	14.8
	Obsessive Compulsive Disorder (OCD)	30	12.3
	Autism Spectrum Disorder (ASD)	30	12.3
	Eating Disorder	17	7.0
	Bipolar Disorder	17	7.0
	Psychosis	8	3.3
	None of above	52	21.4
	(Other) Borderline	7	2.9
	(Other) Oppositional Defiant Disorder	2	0.8

A total of 63.8% of the parents have been called “transphobic” or “bigoted” by their children for one or more reasons, the most common being for: disagreeing with the child about the child’s self-assessment of being transgender (51.2%); recommending that the child take more time to figure out if their feelings of gender dysphoria persist or go away (44.6%); expressing concerns for the child’s future if they take hormones and/or have surgery (40.4%); calling their child by the pronouns they used to use (37.9%); telling the child they thought that hormones or surgery would not help them (37.5%); recommending that their child work on other mental health issues first to determine if they are the cause of the dysphoria (33.3%); calling the child by their birth name (33.3%); or recommending a comprehensive mental health evaluation before starting hormones and/or surgery (20.8%) (Table 10). There were eight cases of estrangement. Estrangement was child-initiated in six cases where the child ran away, moved out, or otherwise refused contact with parent. There were two cases where the estrangement was initiated by the parent because the AYA’s outbursts were affecting younger siblings or there was a threat of violence made by the AYA to the parent.

**Table 10:** Additional behaviors.

		n	%
Parents have been called “transphobic” or “bigoted” by their child for the following reasons*		240	
	Disagreeing with their child about the child’s assessment of being transgender	123	51.2
	Recommending that their child take more time to figure out if their feelings of gender dysphoria persist or go away	107	44.6
	Expressing concerns for their child’s future if the child were to take hormones and/or have surgery	97	40.4
	Referring to their child by the pronouns that they used to use before announcement	91	37.9
	Telling their child that they thought hormones/surgery would not help them	90	37.5
	Calling their child by the child’s birth name	80	33.3
	Recommending that their child work on other mental health issues first to determine if they are the cause of their dysphoria	80	33.3
	Recommending therapy for basic mental health issues (not related to gender)	74	30.8
	Recommending a comprehensive evaluation before starting hormones and/or surgery	50	20.8
	None of the above	87	36.2
Distrust and isolating behaviors exhibited by AYAs*		251	
	Expressed distrust of information about gender dysphoria and transgenderism coming from mainstream doctors and psychologists	130	51.8
	Tried to isolate themselves from their family	124	49.4
	Expressed that they ONLY trust information about gender dysphoria and transgenderism that comes from transgender websites and/or transgender people and sources	117	46.6
	Lost interest in activities where participants aren’t predominantly transgender or LGBTIA	81	32.3
	Lost interest in activities that were not related to transgender or LGBTIA issues	65	25.9
	Stopped spending time with friends who are not transgender	63	25.1
	Expressed distrust of people who are not transgender	57	22.7
	Expressed hostility towards people who are not transgender	46	18.3
	None of the above	44	17.5
Other behavior and outcomes for AYAs*		249	
	Withdrawn from family	112	45.0
	Told other people or posted on social media that their parent is “transphobic”, “abusive”, or “toxic” because the parent does not agree with the child’s assessment of being transgender	107	43.0
	Refused to speak to parent	71	28.5
	Defended the practice of lying to or withholding information from therapists or doctors in order to obtain hormones for transition more quickly	41	16.5
	Tried to run away	17	6.8
	Been unable to obtain a job	25	10.0
	Been unable to hold a job	18	7.2
	Dropped out of college	12	4.8
	Dropped out of high school	12	4.8
	Needed to take a leave of absence from college	12	4.8
	Been fired from a job	9	3.6
	Needed a leave of absence from high school	1	0.4
	None of the above	86	34.5
For any of the above, is this a significant change from the child’s baseline behavior?		161	
	Yes	115	71.4
	No	46	28.6

*may select more than one answer.

AYAs are reported to have exhibited one or more of the following behaviors: expressed distrust of information about gender dysphoria and transgenderism coming from mainstream doctors and psychologists (51.8%); tried to isolate themselves from their family (49.4%); expressed that they only trust information about gender dysphoria and transgenderism that comes from transgender websites and/or transgender people and sources (46.6%); lost interest in activities where participants aren’t predominantly transgender or LGBTIA (32.3%); stopped spending time with friends who were not transgender (25.1%); expressed distrust of people who were not transgender (22.7%) (Table 10). Many AYAs have also: withdrawn from their family (45.0%); told other people or posted on social media that their parent is “transphobic,” “abusive,” or “toxic” because the parent does not agree with child’s self-assessment of being transgender (43.0%); refused to speak to their parent (28.5%), defended the practice of lying to or withholding information from therapists or doctors in order to obtain hormones for transition more quickly (16.5%); tried to run away (6.8%). The behaviors and outcomes listed above were considered significant changes from the child’s baseline behaviors for 71.4% of respondents checking any of the items.

There was a subset of eight cases where parents described watching their child have declining mental well-being as they became gender dysphoric and transgender-identified and then had improving mental well-being as they dropped or backed away from a transgender-identification. One parent described a marked change in her daughter when she was out of school temporarily. “[Her] routine was disrupted. She spent all day on the internet, and lost her many school friends—her only friends were on-line and members of the trans community. In three months, my daughter announced she is trans, gender dysphoric, wants binders and top surgery, testosterone shots…she started self-harming. Now back at school…she tweeted that she’s so young, isn’t sure if she is trans, no longer wants to be referred to by the male name she had chosen…Since she has started back at school and is being exposed to a wide variety of people she is WAY happier.” Another parent described, “My daughter’s insight has improved considerably over the last few years, and she has also outgrown the belief that she is transgender. My daughter actually seemed to be looking for a reason for her depression which is now being successfully treated…My daughter is MUCH happier now that she is being treated for her genuine issues. Coming out as trans made her much worse for a while.”

There was a subset of 30 cases where the AYAs’ transgender-identification occurred in the context of a decline in their ability to function (such as dropping out of high school or college, needing a leave of absence from high school or college, and/or being unable to obtain or hold a job), which parents reported as a significant change from their child’s baseline behavior. The declines were substantial as 43.3% of these AYAs had been identified as academically gifted students (some described as top of their class in high school, earning outstanding grades at prestigious universities) before they began to fail their classes, drop out of high school or college, and became unable to hold a job. In most of these cases (76.7%), there was one or more psychiatric diagnosis made at the same time or within the year (60.0%) or within two years (16.7%) of the AYA’s new transgender-identification. Of the 23 individuals who had a psychiatric diagnosis made within two years of assuming a transgender-identification, 91.3% (21/23) were diagnosed with depression; 73.9% (17/23) with anxiety; 26.0% (6/23) with bipolar disorder; 17.4% (4/23) with borderline personality disorder; 8.7% (2/23) with psychosis/psychotic episode: and 8.7% (2/23) with an eating disorder.

### Clinical encounters

Parents were asked if their child had seen a gender therapist, gone to a gender clinic, or seen a physician for the purpose of beginning transition and 92 respondents (36.2%) answered in the affirmative (Table 11). Many of the respondents clarified that their child had seen a clinician regarding their gender dysphoria for evaluation only. Although participants were not asked directly what kind of provider their child saw, specialties that were mentioned in answers included: general psychologists, pediatricians, family doctors, social workers, gender therapists, and endocrinologists. For parents who knew the content of their child’s evaluation, 71.6% reported that the clinician did not explore issues of mental health, previous trauma, or any alternative causes of gender dysphoria before proceeding and 70.0% report that the clinician did not request any medical records before proceeding. Despite all of the AYAs in this study sample having an atypical presentation of gender dysphoria (no gender dysphoria prior to puberty), 23.8% of the parents who knew the content of their child’s visit reported that the child was offered prescriptions for puberty blockers and/or cross-sex hormones at the first visit.

**Table 11:** Interactions with clinicians.

		n	%
Did the AYA see a gender therapist, go to a gender clinic or see a physician for the purpose of transition?		254	
	No	151	59.4
	Yes	92	36.2
	Don’t know	11	4.3
Did the therapist/physician/clinic staff explore issues of mental health, previous trauma, or any alternative causes of gender dysphoria before proceeding?		100	
	Yes	21	21.0
	No	53	53.0
	Don’t know	26	26.0
Did the therapist/physician/clinic staff request any medical records before proceeding?		99	
	Yes	21	21.2
	No	49	49.5
	Don’t know	29	29.3
Of parents who knew the content of the visit, did the AYA receive an Rx for puberty blockers and/or cross-sex hormones at their first visit?		80	
	AYA received an Rx for puberty blockers and/or cross-sex hormones at their first visit	17	21.2
	AYA was offered a Rx for puberty blockers and/or cross-sex hormones at their first visit, but AYA or parent declined	2	2.5
	Total number of AYAs who received or were offered an Rx at first visit	19	23.8
	AYAs who did not receive/were not offered an Rx at their first visit	61	76.2
Did AYA misrepresent their history to the doctor or relay their history accurately?		96	
	Parent is reasonably sure or positive that their child misrepresented or omitted parts of their history	64	66.7
	Parent is reasonable sure or positive that their child relayed their history completely and accurately	12	12.5
	Don’t know	20	20.8

One participant described, “For the most part, I was extremely frustrated with providers NOT acknowledging the mental disorder, anxiety, depression, etc before recommending hormone replacement therapy.” And two participants described how the clinician treating their child’s gender dysphoria refused to speak with the patients’ primary care physicians. One participant said, “When we phoned the clinic, the doctor was hostile to us, told us to mind our own business. Our family doctor tried to reach our son’s new doctor, but the trans doctor refused to speak with her.” Another respondent shared “The pediatrician/‘gender specialist’ did not return calls or emails from the primary care physician who requested to talk with her about my son’s medical history before she saw and treated him…she disregarded all historical information provided by the family and primary care physician…did not verify any information provided by my…son at his first visit even after being provided with multiple other historical sources which differed significantly from his story.”

When asked about whether their child relayed their history completely and accurately to clinicians or whether they misrepresented or omitted parts of their history, of those who knew the content of their child’s visit, 84.2% of the parent respondents were reasonably sure or positive that their child had misrepresented or omitted parts of their history. Twenty-eight participants provided optional open text responses to this question and the responses were categorized into: describing how the parent knew that the child misrepresented their history (5); the content of what the child misrepresented (6 misrepresenting in general, 4 misrepresenting to the clinician for a total of 10 examples); don’t know/not sure (4); expressing certainty (1); and not relevant (8). For the five participants describing how they knew, the reasons included: being present when it happened, reading the report from the gender specialist, being told by their child that the child had misrepresented the truth, and being informed by the child’s psychiatrist. One respondent shared, “I have read the report from the gender specialist and it omits all the relevant context painting an almost unrecognizable picture of my son.” A second parent simply responded, “I was present.” Another respondent relayed about their (natal male) child, “My daughter told me and her mother that the first therapist she saw asked her stereotypical questions…She was afraid that if she didn’t describe herself as a ‘typical girl’ she would not be believed.” And finally, one respondent wrote, “He has said now that he did [misrepresent his history] and used key words he was advised to say.” Ten participants provided 13 examples of the content of misrepresentations and of these, 6 examples could have been easily verified to be false (claiming to be under the care of a psychiatrist, claiming to be on medication to treat a psychiatric condition, how one was doing academically, and claiming a childhood history of having playmates of one sex when the opposite was observed, and claiming strong childhood preferences for specific toys and clothing that is the opposite of what multiple individuals observed). Three of the content examples would have been challenging to verify as false including: how one was feeling as a child, how one was feeling when a picture was taken, and whether one was from an abusive home. And four of the content examples did not provide enough information to determine if they would be easy or challenging to verify as false, such as “My child distorts her history and our family life on a regular basis,” and “He has created an entire narrative that just isn’t true.”

In addition to the previously mentioned case where the child literally rewrote her history by editing her diary, there were seven respondents who conveyed a process where their child was constantly rewriting their personal history to make it consistent with the idea that they always were transgender and/or had created a childhood history that was not what others had observed. It is unclear whether this process was deliberate or if the individuals were unaware of their actions. The following are quotes describing this phenomenon. One parent said, “…she is actively rewriting her personal history to support the idea that she was always trans.” Another respondent added,”…my daughter denies events I recollect from her childhood and puberty that contradicts her narrative of ‘always knowing she was a boy.’” Another respondent offered, “He is rewriting his personal history to suit his new narrative.” And a fourth respondent described, “[Our] son has completely made up his childhood to include only girl friends and dressing up in girls clothes and playing with dolls, etc. This is not the same childhood we have seen as parents.”

### Qualitative analysis

The open-ended comments from the question about whether the clinician explored mental health, trauma or alternative causes of gender dysphoria before proceeding were selected for qualitative analysis. Nine major themes emerged from the data. Each theme is described in the following paragraphs with supporting quotes from participants.

#### Theme: Failure to explore mental health, trauma or alternative causes of GD

Parents described that clinicians failed to explore their child’s mental health, trauma, or any alternative causes for the child’s gender dysphoria. This failure to explore mental health and trauma occurred even when patients had a history of mental health disorder or trauma, were currently being treated for a mental health disorder, or were currently experiencing symptoms. One participant said, “Nothing other than gender dysphoria was considered to explain my daughter's desire to transition.” Another participant said, “My daughter saw a child therapist and the therapist was preparing to support transgendering and did not explore the depression and anxiety or previous trauma.”

#### Theme: Insufficient evaluation

Another theme was insufficient evaluation where parents described evaluations that were too limited or too superficial to explore mental health, trauma or alternative causes of gender dysphoria. The following are three quotes by three different parents describing insufficient evaluations. One parent said, “The exploration was egregiously insufficient, very shallow, no effort to ask questions, engage in critical thinking about coexisting anxiety, or put on the brakes or even slow down.” Another participant stated, “When we tried to give our son’s trans doctor a medical history of our son, she refused to accept it. She said the half hour diagnosis in her office with him was sufficient, as she considers herself an expert in the field.” And a third parent wrote, “We were STUNNED by the lack of information, medical history sought by therapist and radical treatment suggestion. [One ]visit. The idea is, ‘if they say they were born in the wrong body, they are. To question this will only hurt her and prolong her suffering.’ [Our] daughter has had trauma in [the] past. [She] never was asked about it. [The] therapist did not ask parents a single question about our daughter.”

#### Theme: Unwillingness or disinterest in exploring mental health, trauma or alternative causes of GD

Parents described that clinicians did not seem interested or willing to explore alternative causes. One parent described. “Her current therapist seems to accept her self diagnosis of gender dysphoria and follows what she says without seeming too much interested in exploring the sexual trauma in her past.” Another parent wrote, “The Asperger psychiatrist did not seem to care whether our daughter's gender dysphoria stemmed from Asperger's. If our daughter wanted to be male, then that was enough.” And a third parent said. “The therapist did ask about those issues but seemed to want to accept the idea wholeheartedly that my daughter was transgender first and foremost, all other factors aside.”

#### Theme: Mental health was explored

A few parents had the experience where the clinician either made an appropriate referral for further evaluation or the issues had been addressed previously. One parent said, “[The] previous mental health issues [were] already explored by other therapists ([my] child was in therapy and medicated before coming out as transgender).”

#### Theme: Failure to communicate with patients’ medical providers

Several participants described clinicians who were unwilling to communicate with primary care physicians and mental health professionals even those professionals who were currently treating the patient. One participant relayed, “She did not review the extensive psychiatric records that were available in a shared EMR [electronic medical record] and she did not consult with his outpatient psychiatrist prior to or after starting cross-sex hormonal therapy.” Another parent said, “My child had been seen for mental health issues for several years before presenting this new identity, but the endocrinologist did not consult the mental health professionals for their opinions before offering hormones.”

#### Theme: Misrepresentation of information by the patient

Several participants described how their child misrepresented their history to the clinician, thus, limiting the clinician’s ability to adequately explore mental health, trauma and alternative causes. One participant wrote, “At [the] first visit, [my] daughter's dialogue was well-rehearsed, fabricated stories about her life told to get [the] outcome she desired. She parroted people from the internet.” Another parent reported, “My son concealed the trauma and mental health issues that he and the family had experienced.” And a third parent said, “I overheard my son boasting on the phone to his older brother that ‘the doc swallowed everything I said hook, line and sinker. Easiest thing I ever did.’”

#### Theme: Transition steps were pushed by the clinician

Some parents described clinicians who seemed to push the process of transition before the patient asked for it. One parent described that the doctor gave her daughter a prescription that she didn’t ask for, “The family doctor who gave her the Androgel Rx [prescription] did NOT ask her many questions (she was surprised by this), nor did he await her assessment by a licensed psychiatrist before giving her this Rx. Nor did she ask him for this Rx.” Another parent reported that she and her child were at the endocrinologist’s office only to ask questions, and described, “…[he] didn't listen to a word we were saying. He was too eager to get us set up with a ‘gender therapist’ to get the legal form he needed to start hormones, all while making sure we set up our next appointment within 6 months to start the hormones…”

#### Theme: Parent views were discounted or ignored

Parents describe that the clinicians did not take their concerns seriously. One parent described, “I have to say I don't know, but it is hard to believe that they adequately examined the history of bullying and being ostracized for being different, and the autistic traits that would lend a person like my son to risk everything for identifying with a group. I know that in the few contacts I had with the providers, my concerns were discounted.” And another said, “All of our emails went unanswered and were ignored. We are left out of everything because of our constant questioning of this being right for our daughter [because of her] trauma and current depression, anxiety and self-esteem problems.”

#### Theme: Parent had concerns about the clinicians’ competence, professionalism or experience

Parents expressed doubts about the clinicians regarding their experience, competence or professionalism. One parent said, “The clinic told me they explored these issues. I asked the risk manager at [redacted] if they'd considered a personality disorder. ‘Oh, no,’ she laughed. ‘That's only with the older patients, not the teenagers.’ I'm deeply suspicious of their competence.” Another parent described, “What does concern me is that the people she talked to seemed to have no sense of professional duties, but only a mission to promote a specific social ideology.”

### Steps towards transition and current identification status

This section reports on the duration of AYA transgender-identification (time from the AYA’s announcement of a transgender identity until the time the parent completed the survey) that covers, on average, 15.0 months (range 0.1–120 months) with a median of 11 months (Table 12). The steps taken towards transition during this timeframe are listed in Table 12. At the end of the timeframe, 83.2% of the AYAs were still transgender-identified, 5.5% were not still transgender-identified (desisted), 2.7% seemed to be backing away from transgender-identification, and 8.6% of the parents did not know if their child was still identifying as transgender. Descriptions of backing away or moving from transgender-identified to not transgender-identified include the following. One parent observed, “She identified as trans for six months … Now back at school, she is thinking maybe she's not trans.” Another parent offered, “My daughter [identified] as trans from ages 13–16. She gradually desisted as she developed more insight into who she is.” One parent described that after one year of identifying as transgender, “basically, she changed her mind once she stopped spending time with that particular group of friends.” The duration of transgender-identification of the AYAs who were still transgender-identified at the time of survey was compared to the duration of those who were no longer transgender-identified and those who seemed to be backing away from a transgender-identification (combined) by t-test. The difference between these groups was statistically significant (p = .025), with a t-value of -2.25 showing that those who were no longer transgender-identified and backing away had a longer duration of identification (mean = 24.1 months) and those who were still transgender-identified had a shorter mean duration (mean = 14.4 months).

**Table 12:** Transition steps and disposition.

		n	%
Transition Steps*		256	
	Changed hairstyle	216	84.4
	Changed style of clothing	210	82.0
	Asks to be called a new name	188	73.4
	Asks for different pronouns	175	68.4
	Taken cross-sex hormones	29	11.3
	Legally changed name on government documents	19	7.4
	Taken anti-androgens	11	4.3
	Taken puberty blockers	7	2.7
	Had surgery	5	2.0
	None of the above	14	5.5
Disposition		256	
	Still transgender-identified	213	83.2
	Not transgender-identified any more (desisted)	14	5.5
	Seems to be backing away from transgender-identification	7	2.7
	Parent doesn’t know if the child is still transgender-identified	22	8.6
	De-transitioned (also counted in desisted category)	3	1.2
Duration of transgender-identification overall	Median duration 11 months, Mean duration 15.0 months (range 0.1 months-120 months), median 11 months	225	
Duration of transgender-identification if still transgender-identified	Median duration 11 months, mean duration 14.4 months, range (0.1 months-72 months)	204	
Duration of transgender-identification if no longer transgender-identified	Median duration 12 months, mean duration 24.2 months, range (.75 months to 120 months)	13	
Duration of transgender-identification if backing away	Median duration 12 months, mean duration 15 months, range (3 months-36 months)	8	

*may select more than one answer.

To explore the differences between the AYAs who had exposure to social influence (friend group, internet/social media, or both) and AYAs who did not have a clear exposure to social influence (neither and don’t know), a series of chi-squared calculations were performed for selected variables. (See Table 13.) Statistically significant differences were revealed for AYAs with exposure to social influences having worse outcomes for mental well-being and parent-child relationships, and greater numbers exhibiting distrust, isolating and anti-social behaviors including: narrowed range of interests and hobbies, expressing that they only trusted information from transgender sources, trying to isolate themselves from their family, losing interest in activities that weren’t predominantly with transgender or LGBTIA participants, and telling people or posting on social media that their parent is “transphobic,” “abusive,” or “toxic” because the parent doesn’t agree with the child’s assessment of being transgender. Although the differences in additional isolating and anti-social behaviors did not reach statistical significance, these behaviors trended towards higher rates in the AYAs who were exposed to social influence and may have not reached significant levels due to small numbers. No significant difference for age of AYA (at announcement or at time of survey completion) was detected between groups by a one-way ANOVA.

**Table 13:** chi-squared comparisons for exposure to social influence (SI) vs not exposure to social influence (NSI).

		SI n (%)	NSI n (%)	p
Sex		222	34	.123
	Female	187 (84.2)	25 (73.5)	
	Male	35 (15.8)	9 (26.5)	
**Indicators of childhood GD**		221	33	**.004**
	**0–2 indicators**	**216 (97.7)**	**29 (87.9)**	
	**3–4 indicators**	**5 (2.3)**	**4 (12.1)**	
Currently have two or more GD indicators		214	34	.808
	Yes	179(83.6)	29 (85.3)	
	No	35(16.4)	5(14.7)	
**No mental health or NDD diagnoses before onset of GD**		222	34	**.036**
	**Answered “None of the above”**	**87(39.9)**	**7 (20.6)**	
**Mental well-being since announcement**		220	33	**.001**
	**Worse**	**114 (51.8)**	**6 (18.2)**	
	**Better**	**24 (10.9)**	**8 (24.2)**	
	**Unchanged/Mixed**	**82 (37.3)**	**19 (57.6)**	
**Parent-child relationship since announcement**		219	33	**.006**
	**Worse**	**134 (61.2)**	**11 (33.3)**	
	**Better**	**13 (5.9)**	**5 (15.2)**	
	**Unchanged/Mixed**	**72 (32.9)**	**17 (51.5)**	
**Range of interests and hobbies**		**220**	**34**	**<0.001**
	**Broader range of interests and hobbies**	**10 (4.5)**	**3 (8.8)**	
	**Narrowed range of interest and hobbies**	**139 (63.2)**	**9 (26.5)**	
	**Unchanged range**	**71 (32.3)**	**22 (64.7)**	
Distrust and Isolating Behaviors		222	34	
	**Tried to isolate themselves from family**	**114(51.4)**	**10 (29.4)**	**.017**
	**Expressed that they ONLY trust information about GD and transgenderism that comes from transgender sources**	**107 (48.2)**	**10 (29.4)**	**.041**
	**Lost interest in activities where participants aren’t predominantly transgender or LGBTIA**	**76 (34.2)**	**5 (14.7)**	**.023**
	Stopped spending time with non-transgender friends	59 (26.6)	4 (11.8)	.062
	Expressed distrust of people who are not transgender	52 (23.4)	5 (14.7)	.255
	**Told people or posted on social media that their parent is “transphobic,” “abusive,” or “toxic” because the parent doesn’t agree with the child’s assessment of being transgender**	**102 (45.9)**	**5 (14.7)**	**<0.001**
	Defended the practice of lying to or withholding information from doctors/therapists to get hormones for transition more quickly	38 (17.1)	3 (8.8)	.219
	Brought up the issue of suicide in transgender teens as a reason parents should agree to treatment	55 (24.8)	4 (11.8)	.093
Did the AYA misrepresent their history to the doctor or relay it accurately?		68	8	.075
	Parent is reasonable sure or positive that their child misrepresented or omitted parts of their history	59 (86.8)	5 (62.5)	
	Parent is reasonable sure or positive that child relayed their history completely and accurately	9 (13.2)	3 (37.5)	

## Discussion

This research describes parental reports about a sample of AYAs who would not have met diagnostic criteria for gender dysphoria during their childhood but developed signs of gender dysphoria during adolescence or young adulthood. The strongest support for considering that the gender dysphoria was new in adolescence or young adulthood is the parental answers for DSM 5 criteria for childhood gender dysphoria. Not only would none of the sample have met threshold criteria, the vast majority had zero indicators. Although one might argue that three of the indicators could plausibly be missed by a parent (A1, A7, and A8 if the child had not expressed these verbally), five of the indicators (A2-6) are readily observable behaviors and preferences that would be difficult for a parent to miss. Six indicators (including A1) are required for a threshold diagnosis. The nonexistent and low numbers of readily observable indicators reported in the majority of this sample does not support a scenario in which gender dysphoria was always present but was only recently disclosed to the parents.

Parents reported that before the onset of their gender dysphoria, many of the AYAs had been diagnosed with at least one mental health disorder or neurodevelopmental disability and many had experienced a traumatic or stressful event. Experiencing a sex or gender related trauma was not uncommon, nor was experiencing a family stressor (such as parental divorce, death of a parent, or a mental health disorder in a sibling or parent). Additionally, nearly half were described as having engaged in self-harm prior to the onset of their gender dysphoria. In other words, many of the AYAs and their families had been navigating multiple challenges and stressors before gender dysphoria and transgender-identification became part of their lives. This context could possibly contribute to friction between parent and child and these complex, overlapping difficulties as well as experiences of same-sex attraction may also be influential in the development of a transgender identification for some of these AYAs. Care should be taken not to overstate or understate the context of pre-existing diagnoses or trauma in this population as they were absent in approximately one third and present in approximately two thirds of the sample.

This research sample of AYAs also differs from the general population in that it is predominantly natal female, white, and has an over-representation of individuals who are academically gifted, non-heterosexual, and are offspring of parents with high educational attainment [59–61]. The sex ratio favoring natal females is consistent with recent changes in the population of individuals seeking care for gender dysphoria. Gender clinics have reported substantial increases in referrals for adolescents with a change in the sex ratio of patients moving from predominantly natal males seeking care for gender dysphoria to predominantly natal females [26–28, 62]. Although increased visibility of transgender individuals in the media and availability of information online, with a partial reduction of stigma might explain some of the rise in the numbers of adolescents presenting for care [27], it would not directly explain why the inversion of the sex ratio has occurred for adolescents but not adults or why there is a new phenomenon of natal females experiencing late-onset and adolescent-onset gender dysphoria. The unexpectedly high rate of academically gifted AYAs may be related to the high educational attainment of the parents and may be a reflection of parents who are online, able to complete online surveys and are able to question and challenge current narratives about gender dysphoria and transition. There may be other unknown variables that render academically gifted AYAs susceptible to adolescent-onset and late-onset gender dysphoria. The higher than expected rate of non-heterosexual orientations of the AYAs (prior to announcement of a transgender-identity) may suggest that the desire to be the opposite sex could stem from experiencing homophobia as a recent study showed that being the recipient of homophobic name calling from one’s peers was associated with a change in gender identity for adolescents [63]. The potential relationship of experienced homophobia and the development of a rapid onset of gender dysphoria during adolescence or young adulthood as perceived by parents deserves further study.

This sample is distinctively different than what is described in previous research about gender dysphoria because of the distribution of cases occurring in friendship groups with multiple individuals identifying as transgender, the preponderance of adolescent (natal) females, the absence of childhood gender dysphoria, and the perceived suddenness of onset. In this study, parental reports of transgender identification duration in AYAs suggest that in some cases (~8% in this study) gender dysphoria and transgender-identification may be temporary, and that longer observation periods may be needed to assess such changes. Further research is needed to verify these results. There have been anecdotal reports of adolescents who desisted approximately 9–36 months after showing signs of a rapid onset of gender dysphoria, but longitudinal research following AYAs with gender dysphoria would be necessary to study desistance trends. Although it is still unknown whether transition in gender dysphoric individuals decreases, increases, or fails to change the rates of attempted or completed suicides [64], this study documents AYAs using a suicide narrative as part of their arguments to parents and doctors towards receiving support and transition services. Despite the possibility that the AYAs are using a suicide narrative to manipulate others, it is critical that any suicide threat, ideation or concern is taken seriously and the individual should be evaluated immediately by a mental health professional.

The majority of parents were reasonably sure or certain that their child misrepresented or omitted key parts of their history to their therapists and physicians. In some cases, the misrepresentation of one’s history may simply be a deliberate act by a person who is convinced that transition is the only way that they will feel better and who may have been coached that lying is the only way to get what they think they need. For others, the misrepresentation may not be a conscious act. The creation of an alternate version of one’s childhood that conforms to a story of always knowing one was transgender and that is in sharp contrast to the childhood that was observed by third parties raises the question of whether there has been the creation of false childhood memories as part of, or outside of, the therapy process. Respondent accounts of clinicians who ignored or disregarded information (such as mental health symptoms and diagnoses, medical and trauma histories) that did not support the conclusion that the patient was transgender, suggests the possibility of motivated reasoning and confirmatory biases on the part of clinicians. In the 1990s, the beliefs and practices of many mental health professionals may have contributed to their patients’ creation of false childhood memories consistent with a child sexual abuse narrative and research since then has shown that false childhood memories of mundane events can be implanted in laboratory settings [65–67]. It may be worthwhile to explore if, in today’s culture, there might be beliefs and practices of some mental health professionals that are contributing to their patients’ creation of false childhood memories consistent with an “always knew/always were transgender” narrative.

## Emerging hypotheses

### Hypothesis 1: Social influences can contribute to the development of gender dysphoria

It is unlikely that friends and the internet can make people transgender. However, it is plausible that the following can be initiated, magnified, spread, and maintained via the mechanisms of social and peer contagion: (1) the *belief* that non-specific symptoms (including the symptoms associated with trauma, symptoms of psychiatric problems, and symptoms that are part of normal puberty) should be perceived as gender dysphoria and their presence as proof of being transgender; 2) the *belief* that the only path to happiness is transition; and 3) the *belief* that anyone who disagrees with the self-assessment of being transgender or the plan for transition is transphobic, abusive, and should be cut out of one’s life. The spread of these beliefs could allow vulnerable AYAs to misinterpret their emotions, incorrectly believe themselves to be transgender and in need of transition, and then inappropriately reject all information that is contrary to these beliefs. In other words, “gender dysphoria” may be used as a catch-all explanation for any kind of distress, psychological pain, and discomfort that an AYA is feeling while transition is being promoted as a cure-all solution.

One of the most compelling findings supporting a potential role of social and peer contagion in the development or expression of a rapid onset of gender dysphoria is the clusters of transgender-identification occurring within friendship groups. The expected prevalence of transgender young adult individuals is 0.7% [8]. Yet, according to the parental reports, more than a third of the friendship groups described in this study had 50% or more of the AYAs in the group becoming transgender-identified in a similar time frame. This suggests a localized increase to more than 70 times the expected prevalence rate. This is an observation that demands urgent further investigation. One might argue that high rates of transgender-identified individuals within friend groups may be secondary to the process of friend selection: choosing transgender-identified friends deliberately rather than the result of group dynamics and observed coping styles contributing to multiple individuals, in a similar timeframe, starting to interpret their feelings as consistent with being transgender. More research will be needed to finely delineate the timing of friend group formation and the timing and pattern of each new declaration of transgender-identification. Although friend selection may play a role in these high percentages of transgender-identifying members in friend groups, the described pattern of multiple friends (and often the majority of the friends in the friend group) *becoming* transgender-identified in a similar timeframe suggests that there may be more than just friend selection behind these elevated percentages.

There are many insights from our understanding of peer contagion in eating disorders and anorexia that may apply to the potential role(s) of peer contagion in the development of gender dysphoria. Just as friendship cliques can set the level of preoccupation with one’s body, body image, weight, and techniques for weight loss [37–39], so too may friendship cliques set a level of preoccupation with one’s body, body image, gender, and the techniques to transition. The descriptions of pro-anorexia subculture group dynamics where the thinnest anorexics are admired while the anorexics who try to recover from anorexia are ridiculed and maligned as outsiders [39–41] resemble the group dynamics in friend groups that validate those who identify as transgender and mock those who do not. And the pro-eating-disorder websites and online communities providing inspiration for weight loss and sharing tricks to help individuals deceive parents and doctors [42–44] may be analogous to the inspirational YouTube transition videos and the shared online advice about manipulating parents and doctors to obtain hormones.

### Hypothesis 2: Parental conflict might provide alternative explanations for selected findings

Parents reported subjective declines in their AYAs’ mental health and in parent-child relationships after the children disclosed a transgender identification. Additionally, per parent report, almost half of the AYAs withdrew from family, 28.5% refused to speak to a parent, and 6.8% tried to run away. It is possible that some of these findings might be secondary to parent-child conflict. Parent-child conflict could arise from disagreement over the child’s self-assessment of being transgender. It is also possible that some parents might have had difficulty coping or could have been coping poorly or maladaptively with their child’s disclosure. Other potential explanations for the above findings include worsening of AYAs’ pre-existing (or onset of new) psychiatric conditions or the use of maladaptive coping mechanisms. To further evaluate these possibilities, future studies should incorporate information about family dynamics, parent-child interactions, parent coping, child coping, and psychiatric trajectories. This study did not collect data about the parents’ baseline coping styles, how they were coping with their child’s disclosure, and whether their coping seemed to be maladaptive or adaptive. Nor did it explore parents’ mental well-being. Future studies should explore these issues as well.

Although most parents reported an absence of childhood indicators for gender dysphoria, it is possible that these indicators might have existed for some of the AYAs and that some parents either failed to notice or ignored these indicators when they occurred. Because the readily observable indicators could also have been observed by other people in the child’s life, future studies should include input from parents, AYAs and from third party informants such as teachers, pediatricians, mental health professionals, babysitters, and other family members to verify the presence or absence of readily observable behaviors and preferences during childhood. Parental approaches to their child’s gender dysphoria might contribute to specific outcomes. This study did not specifically explore parental approaches to gender dysphoria or parental views on medical or surgical interventions. Additional studies that explore whether parents support or don’t support: gender exploration; gender nonconformity; non-heterosexual sexual identities; mental health evaluation and treatment; and exploration of potential underlying causes for dysphoria would be extremely valuable. It would also be worthwhile to explore whether parents favor affirming the child as a person or affirming the child’s gender identity and whether parents hold liberal, cautious, or negative views about the use of medical and surgical interventions for gender dysphoria in AYAs.

### Hypothesis 3: Maladaptive coping mechanisms may underlie the development of gender dysphoria for some AYAs

For some individuals, the drive to transition may represent an ego-syntonic but maladaptive coping mechanism to avoid feeling strong or negative emotions similar to how the drive to extreme weight loss can serve as an ego-syntonic but maladaptive coping mechanism in anorexia nervosa [68–69]. A maladaptive coping mechanism is a response to a stressor that might relieve the symptoms temporarily but does not address the cause of the problem and may cause additional negative outcomes. Examples of maladaptive coping mechanisms include the use of alcohol, drugs, or self-harm to distract oneself from experiencing painful emotions. One reason that the treatment of anorexia nervosa is so challenging is that the drive for extreme weight loss and weight loss activities can become a maladaptive coping mechanism that allows the patient to avoid feeling and dealing with strong emotions [69–70]. In this context, dieting is not felt as distressing to the patient, because it is considered by the patient to be the solution to her problems, and not part of the problems. In other words, the dieting and weight loss activities are ego-syntonic to the patient. However, distress is felt by the patient when external actors (doctors, parents, hospital staff) try to interfere with her weight loss activities thus curtailing her maladaptive coping mechanism.

Findings that may support a maladaptive coping mechanism hypothesis include that the most likely description of AYA ability to use negative emotions productively was poor/extremely poor and the majority of AYAs were described as “overwhelmed by strong emotions and tries to/goes to great lengths to avoid experiencing them.” Although these are not validated questions, the findings suggest, at least, that there is a history of difficulty dealing with emotions. The high frequency of parents reporting AYA expectations that transition would solve their problems coupled with the sizable minority who reported AYA unwillingness to work on basic mental health issues before seeking treatment support the concept that the drive to transition might be used to avoid dealing with mental health issues and aversive emotions. Additional support for this hypothesis is that the sample of AYAs described in this study are predominantly female, were described by parents as beginning to express symptoms during adolescence and contained an overrepresentation of academically gifted students which bears a strong resemblance to populations of individuals diagnosed with anorexia nervosa [71–75]. The risk factors, mechanisms and meanings of anorexia nervosa [69–70, 76] may ultimately prove to be a valuable template to understand the risk factors, mechanisms, and meanings for some cases of gender dysphoria.

Transition as a drive to escape one’s gender/sex, emotions, or difficult realities might also be considered when the drive to transition arises after a sex or gender-related trauma or within the context of significant psychiatric symptoms and decline in ability to function. Although trauma and psychiatric disorders are not specific for the development of gender dysphoria, these experiences may leave a person in psychological pain and in search of a coping mechanism. The first coping mechanism that a vulnerable person adopts may be the result of their environment and which narratives for pain and coping are most prevalent in that environment—in some settings a gender dysphoria/drive to transition may be the dominant paradigm, in some settings a body dysphoria/drive for extreme weight loss is dominant, and in another the use of alcohol and drugs to cope with pain may be dominant. Because maladaptive coping mechanisms do not address the root cause of distress and may cause their own negative consequences, an outcome commonly reported for this sample, AYAs experiencing a decline in their mental well-being after transgender-identification, is consistent with this hypothesis. There was a subset of AYAs for whom parents reported improvement in their mental well-being as they desisted from their transgender-identification which would not be inconsistent with moving from a maladaptive coping mechanism to an adaptive coping mechanism.

If the above hypotheses are correct, rapid onset of gender dysphoria that is socially mediated and/or used as a maladaptive coping mechanism may be harmful to AYAs in the following ways: (1) non-treatment or delayed treatment for trauma and mental health problems that might be the root of (or at least an inherent part of) the AYAs’ issues; (2) alienation of the AYAs from their parents and other crucial social support systems; (3) isolation from mainstream, non-transgender society, which may curtail educational and vocational potential; and (4) the assumption of the medical and surgical risks of transition without benefit. In addition to these indirect harms, there is also the possibility that this type of gender dysphoria, with the subsequent drive to transition, may represent a form of intentional self-harm. Promoting the affirmation of a declared gender and recommending transition (social, medical, surgical) without evaluation may add to the harm for these individuals as it can reinforce the maladaptive coping mechanism, prolong the length of time before the AYA accepts treatment for trauma or mental health issues, and interfere with the development of healthy, adaptive coping mechanisms. It is especially critical to differentiate individuals who would benefit from transition from those who would be harmed by transition before proceeding with treatment.

## Reflections

Clinicians need to be aware of the myriad of barriers that may stand in the way of making accurate diagnoses when an AYA presents with a desire to transition including: the developmental stage of adolescence; the presence of subcultures coaching AYAs to mislead their doctors; and the exclusion of parents from the evaluation. In this study, 22.3% of AYAs were reported as having been exposed to online advice about what to say to doctors to get hormones, and 17.5% to the advice that it is acceptable to lie to physicians; and the vast majority of parents were reasonably sure or positive that their child misrepresented their history to their doctor or therapist. Furthermore, although parents may be knowledgeable informants on matters of their own child’s developmental, medical, social, behavioral, and mental health history- and quite possibly *because* they are knowledgeable- they are often excluded from the clinical discussion by the AYAs, themselves. An AYA telling their clinician that their parents are transphobic and abusive may indeed mean that the parents are transphobic and abusive. However, the findings of this research indicate that it is also possible that the AYA calls the parent transphobic and abusive because the parent disagrees with the child’s self-diagnosis, has expressed concern for the child’s future, or has requested that the child be evaluated for mental health issues before proceeding with treatment.

The findings of this study suggest that clinicians need to be cautious before relying solely on self-report when AYAs seek social, medical or surgical transition. Adolescents and young adults are not trained medical professionals. When AYAs diagnose their own symptoms based on what they read on the internet and hear from their friends, it is quite possible for them to reach incorrect conclusions. It is the duty of the clinician, when seeing a new AYA patient seeking transition, to perform their own evaluation and differential diagnosis to determine if the patient is correct or incorrect in their self-assessment of their symptoms and their conviction that they would benefit from transition. This is not to say that the convictions of the patient should be dismissed or ignored, some may ultimately benefit from transition. However, careful clinical exploration should not be neglected, either. The patient’s history being significantly different than their parents’ account of the child’s history should serve as a red flag that a more thorough evaluation is needed and that as much as possible about the patient’s history should be verified by other sources. The findings that the majority of clinicians described in this study did not explore trauma or mental health disorders as possible causes of gender dysphoria or request medical records in patients with atypical presentations of gender dysphoria is alarming. The reported behavior of clinicians refusing to communicate with their patients’ parents, primary care physicians, and psychiatrists betrays a resistance to triangulation of evidence which puts AYAs at considerable risk.

It is possible that some teens and young adults may have requested that their discussions with the clinicians addressing gender issues be kept confidential from their parents, as is their right (except for information that would put themselves or others at harm). However, maintaining confidentiality of the patient does not prevent the clinician from listening to the medical and social history of the patient provided by the parent. Nor does it prevent a clinician from accepting information provided by the patient’s primary care physicians and psychiatrists. Because adolescents may not be reliable historians and may have limited awareness and insight about their own emotions and behaviors, the inclusion of information from multiple informants is often recommended when working with or evaluating minors. One would expect that if a patient refuses the inclusion of information from parents and physicians (prior and current), that the clinician would explore this with the patient and encourage them to reconsider. At the very least, if a patient asks that all information from parents and medical sources be disregarded, it should raise the suspicion that what the patient is presenting may be less than forthcoming and the clinician should proceed with caution.

The argument to surface from this study is not that the insider perspectives of AYAs presenting with signs of a rapid onset of gender dysphoria should be set aside by clinicians, but that the insights of parents are a pre-requisite for robust triangulation of evidence and fully informed diagnosis. All parents know their growing children are not always right, particularly in the almost universally tumultuous period of adolescence. Most parents have the awareness and humility to know that even as adults they are not always right themselves. When an AYA presents with signs of a rapid onset of gender dysphoria it is incumbent upon all professionals to fully respect the young person’s insider perspective but also, in the interests of safe diagnosis and avoidance of clinical harm, to have the awareness and humility themselves to engage with parental perspectives and triangulate evidence in the interest of validity and reliability.

The strengths of this study include that it is the first empirical description of a specific phenomenon that has been observed by parents and clinicians [14] and that it explores parent observations of the psychosocial context of youth who have recently identified as transgender with a focus on vulnerabilities, co-morbidities, peer group interactions, and social media use. Additionally, the qualitative analysis of responses about peer group dynamics provides a rich illustration of AYA intra-group and inter-group behaviors as observed and reported by parents. This research also provides a glimpse into parent perceptions of clinician interactions in the evaluation and treatment of AYAs with an adolescent-onset (or young adult-onset) of gender dysphoria symptoms.

The limitations of this study include that it is a descriptive study and thus has the known limitations inherent in all descriptive studies. This is not a prevalence study and does not attempt to evaluate the prevalence of gender dysphoria in adolescents and young adults who had not exhibited childhood symptoms. Likewise, this study’s findings did not demonstrate the degree to which the onset of gender dysphoria symptoms may be socially mediated or associated with a maladaptive coping mechanism, although these hypotheses were discussed here. Gathering more data on the topics introduced is a key recommendation for further study. It is not uncommon for first, descriptive studies, especially when studying a population or phenomenon where the prevalence is unknown, to use targeted recruiting. To maximize the possibility of finding cases meeting eligibility criteria, recruitment is directed towards communities that are likely to have eligible participants. For example, in the first descriptive study about children who had been socially transitioned, the authors recruited potential subjects from gender expansive camps and gender conferences where parents who supported social transition for young children might be present and the authors did not seek out communities where parents might be less inclined to find social transition for young children appropriate [77]. In the same way, for the current study, recruitment was targeted primarily to sites where parents had described the phenomenon of a rapid onset of gender dysphoria because those might be communities where such cases could be found. The generalizability of the study must be carefully delineated based on the recruitment methods, and, like all first descriptive studies, additional studies will be needed to replicate the findings.

Three of the sites that posted recruitment information expressed cautious or negative views about medical and surgical interventions for gender dysphoric adolescents and young adults and cautious or negative views about categorizing gender dysphoric youth as transgender. One of the sites that posted recruitment information is perceived to be pro-gender-affirming. Hence, the populations viewing these websites might hold different views or beliefs from each other. And both populations may differ from a broader general population in their attitudes about transgender-identified individuals. This study did not explore specific participant views about medical and surgical interventions for gender dysphoric youth or whether participants support or don’t support: exploration of gender identity, exploration of potential underlying causes for gender dysphoria, affirmation of children as valued individuals or affirmation of children’s gender identity. Future studies should explore all these issues. This study cannot speak to those details about the participants.

Respondents were asked, “Do you believe that transgender people deserve the same rights and protections as others in your country?” which is a question that was adapted from a question used for a US national poll [78]. Although this question cannot elicit specific details about a persons’ beliefs about medical interventions, beliefs about transgender identification, or their beliefs about their own child, it can be used to assess if the participants in this study are similar in their basic beliefs about the rights of transgender people to the participants in the US national poll. The majority (88.2%) of the study participants gave affirmative answers to the question which is consistent with the 89% affirmative response reported in a US national poll [78]. All self-reported results have the potential limitation of social desirability bias. However, comparing this self-report sample to the national self-report sample [78], the results show similar rates of support. Therefore, there is no evidence that the study sample is appreciably different in their support of the rights of transgender people than the general American population. It is also important to note that recruitment was not limited to the websites where the information about the study was first posted. Snowball sampling was also used so that any person viewing the recruitment information was encouraged to share the information with any person or community where they thought there could be potentially eligible participants, thus substantially widening the reach of potential respondents. In follow up studies on this topic, an even wider variety of recruitment sources should be attempted.

Another limitation of this study is that it included only parental perspective. Ideally, data would be obtained from both the parent and the child and the absence of either perspective paints an incomplete account of events. Input from the youth would have yielded additional information. Further research that includes data collection from both parent and child is required to fully understand this condition. However, because this research has been produced in a climate where the input from parents is often neglected in the evaluation and treatment of gender dysphoric AYAs, this research supplies a valuable, previously missing piece to the jigsaw puzzle. If Hypothesis 3 is correct that for some AYAs gender dysphoria represents an ego-syntonic maladaptive coping mechanism, data from parents are especially important because affected AYAs may be so committed to the maladaptive coping mechanism that their ability to assess their own situation may be impaired. Furthermore, parents uniquely can provide details of their child’s early development and the presence or absence of readily observable childhood indicators of gender dysphoria are especially relevant to the diagnosis. There are, however, obvious limitations to relying solely on parent report. It is possible that some of the participating parents may not have noticed symptoms of gender dysphoria before their AYA’s disclosure of a transgender identity; could have been experiencing shock, grief, or difficulty coping from the disclosure; or even could have chosen to deny or obscure knowledge of long term gender dysphoria. Readers should hold this possibility in mind. Overall, the 200 plus responses appear to have been prepared carefully and were rich in detail, suggesting they were written in good faith and that parents were attentive observers of their children's lives. Although this research adds the necessary component of parent observation to our understanding of gender dysphoric adolescents and young adults, future study in this area should include both parent and child input.

This research does not imply that no AYAs who become transgender-identified during their adolescent or young adult years had earlier symptoms nor does it imply that no AYAs would ultimately benefit from transition. Rather, the findings suggest that *not all* AYAs presenting at these vulnerable ages are correct in their self-assessment of the cause of their symptoms and *some* AYAs may be employing a drive to transition as a maladaptive coping mechanism. It may be difficult to distinguish if an AYA’s declining mental health is occurring due to the use of a maladaptive coping mechanism, due to the worsening of a pre-existing (or onset of a new) psychiatric condition, or due to conflict with parents. Clinicians should carefully explore these options and try to clarify areas of disagreement with confirmation from outside sources such as medical records, psychiatrists, psychologists, primary care physicians, and other third party informants where possible. Further study of maladaptive coping mechanisms, psychiatric conditions and family dynamics in the context of gender dysphoria and mental health would be an especially valuable contribution to better understand how to treat youth with gender dysphoria.

More research is needed to determine the incidence, prevalence, persistence and desistence rates, and the duration of gender dysphoria for adolescent-onset gender dysphoria and to examine whether rapid-onset gender dysphoria is a distinct and/or clinically valid subcategory of gender dysphoria. Adolescent-onset gender dysphoria is sufficiently different from early-onset of gender dysphoria that persists or worsens at puberty and therefore, the research results from early-onset gender dysphoria should not be considered generalizable to adolescent-onset gender dysphoria. It is currently unknown whether the gender dysphorias of adolescent-onset gender dysphoria and of late-onset gender dysphoria occurring in young adults are transient, temporary or likely to be long-term. Without the knowledge of whether the gender dysphoria is likely to be temporary, extreme caution should be applied before considering the use of treatments that have permanent effects such as cross-sex hormones and surgery. Research needs to be done to determine if affirming a newly declared gender identity, social transition, puberty suppression and cross-sex hormones can cause an iatrogenic persistence of gender dysphoria in individuals who would have had their gender dysphoria resolve on its own and whether these interventions prolong the duration of time that an individual feels gender dysphoric before desisting. There is also a need to discover how to diagnose these conditions, how to treat the AYAs affected, and how best to support AYAs and their families. Additionally, analyses of online content for pro-transition sites and social media should be conducted in the same way that content analysis has been performed for pro-eating disorder websites and social media content [44]. Finally, further exploration is needed for potential contributors to recent demographic changes including the substantial increase in the number of adolescent natal females with gender dysphoria and the new phenomenon of natal females experiencing late-onset or adolescent-onset gender dysphoria.

## Conclusion

Collecting data from parents in this descriptive exploratory study has provided valuable, detailed information that allows for the generation of hypotheses about potential factors contributing to the onset and expression of gender dysphoria among AYAs. Emerging hypotheses include the possibility of a potential new subcategory of gender dysphoria (referred to as rapid-onset gender dysphoria) that has not yet been clinically validated and the possibility of social influences and maladaptive coping mechanisms contributing to the development of gender dysphoria. Parent-child conflict may also contribute to the course of the dysphoria. More research that includes data collection from AYAs, parents, clinicians and third party informants is needed to further explore the roles of social influence, maladaptive coping mechanisms, parental approaches, and family dynamics in the development and duration of gender dysphoria in adolescents and young adults.

## Supporting information

S1 Appendix Survey instrument. (PDF)

S2 Appendix COREQ checklist. (PDF)
