# Correction: Parent reports of adolescents and young adults perceived to show signs of a rapid onset of gender dysphoria

**DOI:** 10.1371/journal.pone.0214157

**Published:** 2019-03-19

**Authors:** Lisa Littman

## Notice of republication

After publication of this article [1], questions were raised that prompted the journal to conduct a post-publication reassessment of the article, involving senior members of the journal’s editorial team, two Academic Editors, a statistics reviewer, and an external expert reviewer. The post-publication review identified issues that needed to be addressed to ensure the article meets *PLOS ONE*’s publication criteria. Given the nature of the issues in this case, the *PLOS ONE* Editors decided to republish the article, replacing the original version of record with a revised version in which the author has updated the Title, Abstract, Introduction, Discussion, and Conclusion sections, to address the concerns raised in the editorial reassessment. The Materials and methods section was updated to include new information and more detailed descriptions about recruitment sites and to remove two figures due to copyright restrictions. Other than the addition of a few missing values in Table 13, the Results section is unchanged in the updated version of the article. The Competing Interests statement and the Data Availability statement have also been updated in the revised version. The original version of the published article is appended to this Correction as S1 File.

This Correction Notice serves to provide additional clarifications and context for the article in response to questions raised during the post-publication review of this work.

## Emphasis that this is a study of parental observations which serves to develop hypotheses

This study of parent observations and interpretations serves to develop the hypotheses that rapid-onset gender dysphoria is a phenomenon and that social influences, parent-child conflict, and maladaptive coping mechanisms may be contributing factors for some individuals. Rapid-onset gender dysphoria (ROGD) is not a formal mental health diagnosis at this time. This report did not collect data from the adolescents and young adults (AYAs) or clinicians and therefore does not validate the phenomenon. Additional research that includes AYAs, along with consensus among experts in the field, will be needed to determine if what is described here as rapid-onset gender dysphoria (ROGD) will become a formal diagnosis. Furthermore, the use of the term, rapid-onset gender dysphoria should be used cautiously by clinicians and parents to describe youth who appear to fall into this category. The term should not be used in a way to imply that it explains the experiences of all gender dysphoric youth nor should it be used to stigmatize vulnerable individuals. This article has been revised to better reflect that these parent reports provide information that can be used to develop hypotheses about factors that may contribute to the onset and/or expression of gender dysphoria among this demographic group.

Because this is a study of parent reports, there is some information about the AYAs that the parents would not have access to and the answers might reflect parent perspectives. Examples where parent answers reflect their perspective of the AYA include answers concerning the child’s mental well-being, the parent-child relationship, and whether the child has high expectations about transitioning. However, it is also important to note that there are other survey items where the parent would have direct access to information about their child and that those answers reflect items that can be directly observed. Examples of this type include age, natal sex, diagnoses given by medical providers in the presence of the parent, directly observed behaviors of the child and the child’s friend group, school performance, whether the child has dropped out or required a leave of absence from school, has been unable to hold a job, whether the child went to a clinic, or received treatment. Readers are reminded to keep in mind that this is a study of parent report and consideration of what information parents may or may not have access to is an important element of the findings.

## Questions on whether the article describes adolescent-onset gender dysphoria or if it describes something new

There is some controversy over whether what is described as rapid onset of gender dysphoria, particularly in natal females, falls under the existing definition of late-onset or adolescent-onset gender dysphoria or whether it represents a new kind of development or presentation. This controversy might be a false dichotomy because both might be true. Although recent observations of adolescents and young adults who are predominantly natal female having a sudden onset of gender dysphoria symptoms beginning during or after puberty might technically fall under the existing definitions and criteria for adolescent and adult gender dysphoria [2], the substantial change in the demographics of patients presenting for care, the inversion of the sex ratio with disproportionate increase in adolescent natal females [3–5], and the new phenomenon of natal females exhibiting adolescent-onset and late-onset gender dysphoria [6–8] signal that something new may be happening as well. These changes may indicate that there are new etiologies leading to gender dysphoria and it is unclear, particularly without research about these new populations, whether gender dysphoria in this context has the same outcomes, desistence and persistence rates, and response to treatment as the gender dysphorias that have been previously studied.

## Expanded discussion of qualitative analyses

Because this is a descriptive, exploratory study into a new topic with very little existing data, the addition of the qualitative analysis of two questions in addition to the quantitative analysis allowed for a greater depth of information to be used in the development of hypotheses. A grounded theory approach was selected as the strategy of choice for handling the qualitative data. There were two reviewers consisting of a professor with a PhD degree and expertise in qualitative methods (MM) [9] and the author (LL) who holds an MD and MPH degree, and has published both qualitative and quantitative research papers [10–11]. Each reviewer independently read and re-read the open-text responses in an iterative process to identify major themes arising from the data. Once each reviewer independently listed major themes and coded the open-text responses according to those themes, both reviewers compared notes to collaboratively revise and refine the major themes identified. Once an agreed-upon final list of themes was developed, attention was turned back to the data to code the open-text response with the final list of themes. After this task was completed, LL selected salient quotes to reflect each major theme, shared the quotes with MM, and both discussed collaboratively until agreement for the final list of major themes and associated quotes was reached. The incorporation of both the qualitative and quantitative analysis allowed for a more vivid picture of parent perspectives about the friendship group dynamics and behaviors and clinician interactions than could have been obtained from just one type of analysis.

## Clarification of study design, methods, and related limitations

As mentioned in the article, the study design of this research falls under descriptive research: as such, it did not assign an exposure, there were no comparison groups, and the study’s output was hypothesis-generating rather than hypothesis-testing [12]. Descriptive studies often represent a first inquiry into an area of research and the findings of descriptive studies are used to generate new hypotheses that can be tested in subsequent research [12–13]. Because of the known limitations of descriptive studies, claims about causal associations cannot be made [12], and there were none made in the article. The conclusions of the current study are that the findings raise certain hypotheses and that more research is needed. Simple descriptive metrics to describe the quantitative characteristics of a sample in a descriptive study are the appropriate measures to use in this study. Additionally, because the data were collected at one point in time, no claims of cause and effect can be made.

All research methods have advantages and limitations. Obtaining information from parents (and guardians) about the health and well-being of children and adolescents is an established method of research [14]. Parental report, used elsewhere and in this study, offers the advantages of collecting data from adults who are knowledgeable about the child, who are able and willing to complete research activities such as detailed surveys, and who can provide details that are not available by other methods. Limitations of parental report include information that parents may not be aware of and parental biases. Anonymous surveys, used elsewhere and in this study, are advantageous for topics that might be stigmatized and can allow participants to be more honest in their responses but introduce the limitation that the researcher cannot verify the identity and experiences of the participants. The use of targeted recruitment and convenience samples, used elsewhere and in this study, offers the benefit of connecting with hard-to-reach populations but introduces limitations associated with selection bias that can subsequently be addressed by further studies. For the current study, selection bias may have resulted in findings that are more positive or more negative than would be found in a larger and less self-selected population. Subsequent studies should address these issues.

## Updated Information about recruitment

Concerns were raised that this study only posted links to the recruitment information on selected sites that are viewed as being unsupportive of transition. However, announcements about the study included requests to distribute the recruitment information and link, and because information about where the participants encountered the announcement was not collected, it is not known which populations were ultimately reached. It has come to light that a link to the recruitment information and research survey was posted on a private Facebook group perceived to have a pro-gender-affirming perspective during the first week of the recruitment period (via snowball sampling). This private Facebook group is called “Parents of Transgender Children” and has more than 8,000 members. This means that parents participating in this research may have viewed the recruitment information from one of at least four sites with varied perspectives. Specifically, three of the sites that posted recruitment information expressed cautious or negative views about medical and surgical interventions for gender dysphoric adolescents and young adults and cautious or negative views about categorizing gender dysphoric youth as transgender. And, one of the sites that posted recruitment information is perceived to be pro-gender-affirming. The rest of the Correction notice will refer to recruitment from the four sites that are known to have posted the survey in the first week of recruitment: 4thwavenow, transgendertrend, Youth Trans Critical Professionals, and Parents of Transgender Children.

## Parental approaches to gender dysphoria and views on medical interventions

To oversimplify parental approaches as simply “accepting” or “rejecting” misrepresents the range of responses and complexity of approaches that parents take when addressing the needs of their gender dysphoric children. Parental approaches are complex and cover many variables. For example, one parental approach might be to affirm the child as a person, support gender nonconformity, support gender exploration, support mental health evaluation and treatment as needed, support the exploration of potential underlying causes for the dysphoria while expressing caution about medical interventions. Another approach might be to affirm the child’s newly declared gender identity, support gender nonconformity, support a liberal approach to medical intervention while expressing caution about mental health evaluation and caution about the exploration of potential underlying causes for the dysphoria. To categorize the former as “rejecting” and the latter as “accepting” would be inaccurate.

This study recruited participants based on whether participants thought their child exhibited a sudden or rapid onset of gender dysphoria beginning during or after puberty and did not recruit based on parental beliefs about what types of approaches toward gender dysphoric AYAs are best. Although one of the sites posting recruitment information might be considered to hold a pro-gender affirming perspective and three sites might be considered to hold a cautious or even negative perspective about medical or surgical interventions, the site where a participant first heard about the study may not be an accurate reflection of their beliefs and whether they endorse or disagree with the content of the websites. Data about where participants first heard about this study were not collected. Future studies should seek a wider array of websites to post recruitment information, recruit from clinicians with varied approaches to gender dysphoria, and ask specific questions about parental beliefs regarding their approach to their child’s gender dysphoria, including: whether parents support or don’t support gender exploration, gender nonconformity, mental health evaluation and treatment, exploration of potential underlying causes for dysphoria, non-heterosexual sexual identity, and whether they hold a liberal, cautious or negative view about the use of medical and surgical interventions for gender dysphoric youth. Exploration about what types of affirmation are endorsed by parents including affirmation of the child as a person and affirmation of the child’s gender identity would also be valuable.

## Expanded discussion about limitations and biases

Regarding the reporting of gender dysphoria, an absence of childhood gender dysphoria and whether the AYA was gender dysphoric at the time of survey completion were based on parent report of whether certain indicators of gender dysphoria were observed prior to puberty or at the time of the survey. These determinations were not diagnoses made by clinicians. Three of the indicators listed in the DSM-5 include information that a parent might not have access to (unless the child told them directly) [2], and therefore answers based on parent perceptions may not accurately reflect the experiences or traits of the AYAs themselves. However, the other five indicators include readily observable behaviors and preferences that would seem difficult for a parent not to notice such as: strong preference or strong resistance to wearing certain kinds of clothing; strong preference or strong rejection of specific toys, games and activities; and strong preference for playmates of the other gender [2]. It is possible that a parent could have ignored some of these indicators, though other people in the child’s life may have observed them. To improve the reliability of this measure, future studies should include evaluation from clinicians with input from parents, AYAs and from third party informants such as teachers, pediatricians, mental health professionals, babysitters, and other family members who knew the youth during childhood to verify the whether the readily observable behaviors and preferences were present or absent during childhood.

For a clinician to make a diagnosis of gender dysphoria in childhood, a child would need to exhibit at least six of the eight indicators. Given that 97.6% of the participants reported 2 or fewer readily observable indicators, even if hypothetically all participants incorrectly under-reported all three of the subtler indicators, 97.6% would still have fewer than six indicators. So, although no clinical evaluation was performed and a clear presence or absence of a diagnosis cannot be verified, given the reports of the easily observed behaviors and preferences, it can be said that it would be very unlikely for these AYAs to have met criteria for childhood gender dysphoria if they had seen a clinician for an evaluation.

There is expected variation in how objective parents can be about their own children. Some individual biases may limit the objectivity of parents. This descriptive study was not designed to explore or measure the objectivity of participants. Participants may have first learned about this study from one of four (or more) sites described previously where recruitment information was posted. It is possible that exposure to websites that take a cautious or negative approach to transition during adolescence and young adulthood and exposure to websites that take a pro-gender-affirming approach might influence how parents report about their children’s experiences. There have not been any studies to determine if parents who seek information from online sites in general, don’t seek information from online sites, or seek information from specific online sites, including the four sites noted for this study, differ in their ability to provide objective assessments of their children. However, if there were an excess of participants who, compared to other parents who take surveys reporting on their children, were less able to be objective about their children, it could limit some of the findings of the study, particularly for findings that are more interpretive rather than the findings that are more concrete.

The research survey did not specifically ask whether parents supported their AYAs’ exploration of gender identity, so whether and what numbers of participants supported their child’s exploration of gender identity is unknown. However, if there were an excess of parents who did not support the exploration of gender identity, it could potentially result in higher reports of declining mental health. The parents’ perception that their child’s mental health and the parent-child relationship were worse after the child announced a transgender-identification could be due to several variables such as conflict between parent and child, maladaptive coping mechanisms, or worsening psychiatric issues unrelated to gender. The trajectories for adolescent-onset gender dysphoria are not well understood and additional research is desperately needed.

There are many ways that parents can provide support for their child which include: affirming them as a unique and valuable person and as a loved member of the family; supporting their emotional and financial needs; supporting them in pursuing their interests; supporting them to develop the skills needed for self-sufficiency; supporting their choices of gender nonconforming clothing and interests; supporting their exploration of their identity; and supporting them in their critical thinking skills. Parental support is multifaceted and should not be oversimplified into a binary of whether a parent agrees or disagrees with a specific medical course. This study was not designed to measure different types of support provided by parents or levels of support. If there were an excess of parents who were unsupportive of their children, it might affect some of these initial findings. The nature and extent of parental support—including the many different ways that parents can support their children in becoming healthy, self-sufficient adults—is well worth further study.

## Clarification of Fig 1

The purpose of Fig 1 was to provide the reader with a quick sense of what kinds of advice can be found and shared on Reddit and Tumblr. One example includes an excerpt from a publicly available Tumblr blog that posted a list of purported indirect signs of gender dysphoria. This excerpt is indeed an example of advice that can be found on Tumblr. Note, however, that the excerpted Tumblr post itself does not reflect the full content of the original blog it refers to, nor does the excerpt in Fig 1. The original blog is titled, “‘That was dysphoria?’ 8 signs and symptoms of indirect gender dysphoria” [15].

## Discussion of the ICD-11 change from “gender dysphoria” to “gender incongruence”

The ICD-11 will go into effect in January 2022, and, with this change, the new diagnosis of “gender incongruence” will replace “gender dysphoria.” Because the current descriptive, exploratory study raises hypotheses about factors that may contribute to the onset and/or expression of gender dysphoria and concludes that more research is needed, it is unlikely that the change in diagnostic criteria will appreciably change the conclusion of the study, although the terminology may become outdated.

## Supporting information

S1 File PDF of the original article version that was published on August 16, 2018 (two figures removed due to copyright restrictions). (PDF)
