# Supplementary material for: Parent reports of adolescents and young adults perceived to show signs of a rapid onset of gender dysphoria
Source: PLoS One. 2018 Aug 16;13(8):e0202330. doi: 10.1371/journal.pone.0202330 (PMC6095578; doi:10.1371/journal.pone.0202330)
Supplement: S1 Appendix — (PDF) [file pone.0202330.s001.pdf]

Rapid onset gender dysphoria, social media, and peer groups

1. Description and Consent

The purpose of this research study is to gather information to describe an unusual presentation of gender dysphoria. This research study is being conducted by Lisa Littman, MD, MPH, Adjunct Assistant Professor, Icahn School of Medicine at Mount Sinai.

Online, more than 20 individual parent accounts have described seeing their child develop a rapid onset of gender dysphoria beginning in adolescence in the context of increased social media/Internet use and/or being part of a peer group in which one or multiple friends have developed gender dysphoria and come out as transgender during a similar time frame. Several parents have described situations where entire friend groups became gender dysphoric. This presentation is not consistent with what is known about gender dysphoria. We feel that this phenomenon needs to be described and studied scientifically.

You are being asked to take part in a research study if you have a child who, when they were between the ages of 10 and 21, developed a sudden or rapid onset of gender dysphoria. This may have occurred in the context of either increased social media/Internet use, and/or belonging to a friend group in which one or multiple friends have developed gender dysphoria and come out as transgender during a similar time frame.

Being in a research study is completely voluntary. You can choose not to be in this research study. If you agree to take part in this research, you will be asked to complete a confidential, anonymous, web-based survey. Your participation in this study will take about 30-60 minutes. We expect that 10-1000 people will take part in this research study.

You can choose not to answer any question you do not wish to answer, with the exception of one question to determine if you are eligible to proceed with the survey. You can also choose to stop taking the survey at any time before submitting it. You must be at least 18 years old to participate. If you are younger than 18 years old, please stop now.

The possible risks to you in taking part in this research include feeling uncomfortable answering questions or potential loss of confidentiality. To protect your identity as a research subject we will not ask or collect any personal information that could identify you and we will not collect IP addresses. Please do NOT write any identifying information (names, addresses, email addresses, etc) into the free text boxes.

If you have more than one child who fits the above description, please fill out one survey per child.

If you have any questions about this research, please contact Dr. Littman at (347) 443-2215. You may also call the Program for the Protection of Human Subjects Office at the Icahn School of Medicine at Mount Sinai at 212-824-8200.

ELECTRONIC CONSENT: Clicking on the "AGREE" button below indicates that:

• you have read the above information

• you voluntarily agree to participate

• you are at least 18 years of age

* 1. I have read the above information, I voluntarily agree to participate, I am at least 18 years of age:

- ◯ AGREE
- ◯ DISAGREE

2. Welcome to this survey about rapid onset gender dysphoria.

Thank you for participating. Your feedback is important.

If you have more than one child who has had a sudden or rapid onset of gender dysphoria, please complete more than one survey (using one survey to describe one child, a second survey to describe a second child, etc).

For the purpose of the survey, I am going to define a few terms:

Gender Dysphoria (GD) refers to a persistent discomfort with one's biologic sex or assigned gender.

Transgender describes an individual whose gender identity (subjective sense of maleness or femaleness or other) is different than their biological (physical, anatomical, genetic) sex or assigned gender. Transgender is often used as an umbrella term to include several identities such as gender fluid or gender non binary.

The phrases "coming out as transgender" or "announcing that they were transgender" are used interchangeably in the survey to describe the point in time that your child told you or others that they believed that they were transgender.

The term "your child" to refer to children of all ages, including adolescents and young adults.

3.

2. What is your age?

3. What is your sex?

- ◯ Male
- ◯ Female
- ◯ Other (please specify)

4. What is your race/ethnicity? (Check all that apply)

- ☐ Asian
- ☐ Black
- ☐ Hispanic
- ☐ Multiracial
- ☐ White
- ☐ Other (please specify)

5. What is the highest level of school you have completed or the highest degree you have received?

- ◯ Less than a high school degree
- ◯ High school degree or equivalent (e.g., GED)
- ◯ Some college or university but no degree
- ◯ Associate degree
- ◯ Bachelor degree
- ◯ Graduate degree
- ◯ Other (please specify)

6. In what country do you live?

- ◯ United States (US)
- ◯ United Kingdom (UK)
- ◯ Canada
- ◯ Other (please specify)

7. If you live in the United States, in which region do you live?

- ◯ I don't live in the United States
- ◯ Northeast
- ◯ Southeast
- ◯ Mid-Atlantic
- ◯ Midwest
- ◯ Northwest
- ◯ Southwest
- ◯ Other (please specify)

8. How many of your children have experienced a sudden or rapid onset of gender dysphoria, which began after puberty?

- ◯ 0
- ◯ 1
- ◯ 2
- ◯ 3
- ◯ Other (please specify)

9. Do you strongly favor, favor, oppose, or strongly oppose allowing gay and lesbian couples to marry legally?

- ◯ Strongly favor
- ◯ Favor
- ◯ Oppose
- ◯ Strongly Oppose
- ◯ Don't Know

10. More gay and lesbian couples are raising children. Which of the following statements best reflects your opinion?

- ◯ More gay and lesbian couples raising children is generally a good thing for society.
- ◯ More gay and lesbian couples raising children is generally a bad thing for society.
- ◯ More gay and lesbian couples raising children doesn't make much difference for society.
- ◯ Don't know

11. Do you believe that transgender people deserve the same rights and protections as other individuals in your country?

- Yes
- No
- Don't Know
- Other (please specify)

12. Would you support or oppose a law to protect transgender people from discrimination in employment and housing?

- Support
- Oppose
- Don't Know
- Other (please specify)

* 13. Did your child have a sudden or rapid onset of gender dysphoria?

- Yes
- No
- Other (please specify)

Rapid onset gender dysphoria, social media, and peer groups

4.

14. Along with a sudden or rapid onset of gender dysphoria, did your child also?

- ◯ have an increase in their social media/Internet use
- ◯ belong to a friend group in which one or multiple friends became gender dysphoric and came out as transgender during a roughly similar time frame as your child
- ◯ Both
- ◯ Neither

15. When did your child's gender dysphoria begin?

- ◯ Before puberty
- ◯ During puberty
- ◯ After puberty
- ◯ Other (please specify)

16. Can you pinpoint a time when your child seemed not at all gender dysphoric to you? How long between that time and when they came out as transgender?

Please describe

17. How old is your child now?

18. How old was your child when he/she announced that they were transgender?

19. What was your child's biological sex at birth? (Note: biological sex at birth is also called "natal sex")

- ◯ Male
- ◯ Female
- ◯ Other (please specify)

20. Where was your child living when they became gender dysphoric?

- ◯ At home (living with one or both parents)
- ◯ At a boarding school
- ◯ At a college or university
- ◯ Other (please specify)

21. Did your child's announcement that they were transgender come "out of the blue," without significant prior evidence of gender dysphoria?

- ◯ Yes
- ◯ No
- ◯ Other

If you would like to add more information, you may do so here.

22. Do you think your child is CORRECT in their belief that they are transgender?

- ◯ Yes
- ◯ No
- ◯ I don't know
- ◯ Other (please specify)

23. How would you rate your child's overall insight about topics not related to gender?

- ◯ My child's overall insight about topics not related to gender is excellent.
- ◯ My child's overall insight about topics not related to gender is good.
- ◯ My child's overall insight about topics not related to gender is fair or mixed.
- ◯ My child's overall insight about topics not related to gender is poor.
- ◯ My child's overall insight about topics not related to gender is extremely poor.
- ◯ I don't know.

If you would like to add more information, you may do so here.

24. How would you rate your child’s ability to understand their own emotions? (Note, an example of excellent ability would be if your child expressed that while they were happy for a friend who won an award, that they are also disappointed and a little envious because they wanted to be the one to win the award. An example of extremely poor ability would be if a child was sad and had no idea why they were feeling sad.)

- ◯ My child’s ability to understand their own emotions is excellent.
- ◯ My child’s ability to understand their own emotions is good.
- ◯ My child’s ability to understand their own emotions is fair.
- ◯ My child’s ability to understand their own emotions is poor.
- ◯ My child’s ability to understand their own emotions is extremely poor.
- ◯ I don't know.

25. How would you rate your child’s ability to deal with their negative emotions and channel them into something productive? (Note: An example of excellent ability in this area would be taking the emotion of frustration from earning a low grade on a test at school and channeling it into preparing and studying better for the next test. Examples of extremely poor ability would be responding to the emotion of frustration from a low test grade by ignoring it, immersing themselves in computer games to distract themselves, throwing a tantrum, blaming the teacher, calling themselves stupid, avoiding the class, using alcohol or drugs to take their mind off of it, or engaging in self-harm to distract themselves, etc .)

- ◯ My child’s ability to deal with their negative emotions productively is excellent.
- ◯ My child’s ability to deal with their negative emotions productively is good.
- ◯ My child’s ability to deal with their negative emotions productively is fair.
- ◯ My child’s ability to deal with their negative emotions productively is poor.
- ◯ My child’s ability to deal with their negative emotions productively is extremely poor.
- ◯ I don't know.

26. How does your child handle strong emotions? (Please select the best answer).

- ◯ My child is overwhelmed by strong emotions and goes to great lengths to avoid feeling them.
- ◯ My child is overwhelmed by strong emotions and tries to avoid feeling them.
- ◯ My child neither avoids nor seeks out strong emotions.
- ◯ My child tries to seek out situations in order to feel strong emotions.
- ◯ My child goes to great lengths to seek out situations in order to feel strong emotions.
- ◯ None of the above
- ◯ I don't know.

27. Before the onset of puberty, did your child express the following? (Check all that apply)

- Strong desire to be the other gender
- Strong preference for dressing as the other gender
- Strong preference for cross-gender roles in make believe play
- Strong preference for toys stereotypically used by the other gender
- Strong preference for playmates of the other gender
- Rejection of typically masculine (if a boy) or typically feminine (if a girl) toys, games and activities
- Strong dislike of their sexual anatomy
- Strong desire for physical attributes that match the other gender
- None of the above
- Other (please specify)

28. If you checked any items in the last question, were they associated with clinically significant distress or impairment in social, school or other important areas of functioning?

- N/A as my child did not express any of the items in the last question.
- Yes
- No

If you would like to add more information, you may do so here.

29. Currently, is your child expressing any of the following? (Check all that apply) Note: For the purpose of this question, the term "opposite gender" can include any gender that is not consistent with their biological sex.

- Stating that their gender is different than what their body is
- Strong desire to be rid of the body parts/ sex characteristic of their biological sex
- Strong desire to possess the body parts/sex characteristics of the opposite sex
- Strong desire to be the opposite gender
- Strong desire to be treated as the opposite gender
- Strong conviction that they have the typical feelings and reactions of the opposite gender
- None of the above
- Other (please specify)

30. If you checked any items in the last question, were they associated with clinically significant distress or impairment in social, occupational, school or other important areas of functioning?

- N/A as my child did not express any of the items in the last question.
- Yes
- No

If you would like to add more information, you may do so here.

31. In the period of time PRIOR to announcing he or she was transgender, did your child change their gender presentation (for example, did your daughter ask to have her hair cut short and start wearing boyish clothes? Did your son ask to grow his hair long, wear make-up, or dresses?)

- Yes
- No
- I don't know

If you would like to add more information, you may do so here.

32. If you answered "yes" to the previous question, how long BEFORE the time they announced they were transgender did this change occur?

- My child did not change their gender presentation before announcing that they were transgender.
- more than one year
- 6 months to a year
- 3 to 6 months
- 2 months
- 1 month
- less than one month
- less than one week
- Other (please specify)

33. Please check all that describe your child.

- [ ] During childhood, my child did NOT have gender dysphoria or cross-gender behaviors.
- [ ] During puberty, my child did NOT have gender dysphoria or cross-gender behaviors.
- [ ] For most of my child's grade school (ages 7-12), he/she felt excluded by his/her peers.
- [ ] My child had persistent experiences of being bullied before the onset of gender dysphoria.
- [ ] During adolescence, my child had social anxiety.
- [ ] During adolescence, my child had depression.
- [ ] My child has a history of being isolated (not interacting with peers outside of school activities).
- [ ] My child had long periods of time not attending school (due to illness, mental illness, or refusal to attend school).
- [ ] My child had difficulty interacting with their peers.
- [ ] If you would like to add more information, you may do so here.

34. Does your child have very high expectations that transitioning to another gender will solve their problems in social, academic, occupational, or mental health areas?

- Yes
- No
- I don't know

If you would like to add more information, you may do so here.

35. Has your child been part of a friend group where one or more friends has come out as transgender around a similar time frame as your child did?

- Yes
- No
- I don't know

If yes, please describe, noting approximately how many kids were part of this friend group in total.

36. How old was your child when the first friend of the group came out as transgender?

- Not applicable
- less than ten years of age
- 10 years old
- 11 years old
- 12 years old
- 13 years old
- 14 years old
- 15 years old
- 16 years old
- 17 years old
- 18 years old
- 19 years old
- 20 years old
- 21 years old
- Other (please specify)

37. Approximately, how many friends in this group came out as transgender BEFORE your child did?

If you would like to add more information, you may do so here.

38. Approximately, how many friends in this group came out as transgender AFTER your child did?

If you would like to add more information, you may do so here.

39. Did the majority of the friend group become transgender?

- Not applicable
- Yes
- No
- Not sure

If you would like to add more information, you may do so here.

40. Please describe what kind of response your child received from their friend group when they came out as transgender?

- ◯ My child's popularity increased within the friend group when they came out as transgender.
- ◯ My child's popularity decreased within the friend group when they came out as transgender.
- ◯ My child's popularity was unchanged within the friend group when they came out as transgender.
- ◯ I don't know.

If you would like to add more information, you may do so here.

41. To your knowledge, do the friends in this friend group mock, tease, or make fun of people who are not transgender or not LGBTIA? For example, do they make jokes about straight or cisgendered people or post comments about how to put straight or cis-gendered people in their place? If yes, please describe.

- ◯ Yes
- ◯ No
- ◯ I don't know

If you answered yes, please describe.

42. Has your child done any of the following? (Check all that apply)

- ☐ Expressed distrust of people who are not transgender
- ☐ Expressed distrust of information about gender dysphoria and trangenderism coming from mainstream doctors and psychologists
- ☐ Expressed that they ONLY trust information about gender dysphoria and transgenderism that comes from transgender websites and/or transgender people and sources
- ☐ Expressed hostility towards people who are not transgender
- ☐ Stopped spending time with friends who are not transgender
- ☐ Lost interest in activities in which participants are not predominantly transgender or LGBTIA
- ☐ Lost interest in activities in which participants aren't only transgender people
- ☐ Lost interest in activities that were not related to transgender and LGBTIA issues
- ☐ Lost interest in activities that were not related to transgender-only issues
- ☐ Tried to isolate themselves from their family
- ☐ None of the above
- ☐ If you would like to add more information, you may do so here.

43. How would you rate your child’s range of interests and hobbies since becoming gender dysphoric/coming out as transgender? (Note: broadened interests and hobbies means that a child has more interests and hobbies than they used to. Narrowed interests and hobbies means that they have fewer interests and hobbies that they used to).

- ◯ My child’s range of interests and hobbies is much broader.
- ◯ My child’s range of interests and hobbies is somewhat broader.
- ◯ My child’s range of interests and hobbies is basically unchanged.
- ◯ My child’s range of interests and hobbies is somewhat narrower.
- ◯ My child’s range of interests and hobbies is much narrower.
- ◯ There are very few topics outside of transgender issues that my child is still interested in.
- ◯ Don't know

44. BEFORE announcing that they were transgender, what sexual orientation(s) did your child identify as? (Check all that apply)

- ☐ My child did not express any sexual orientation
- ☐ Straight
- ☐ Gay
- ☐ Lesbian
- ☐ Bisexual
- ☐ Pansexual
- ☐ Asexual
- ☐ Prefer not to answer
- ☐ Other (please specify)

45. AFTER announcing that they were transgender, what sexual orientation(s) did your child identify as? (Check all that apply)

- ☐ My child did not express a sexual orientation
- ☐ Straight
- ☐ Gay
- ☐ Lesbian
- ☐ Bisexual
- ☐ Pansexual
- ☐ Asexual
- ☐ Prefer not to answer
- ☐ Other (please specify)

46. Has your child ever dated or had a romantic relationship with another person?

- Yes
- No
- I don't know

47. Did your child go through a progression of several gender identities and sexual orientation identities? (For example, going from defining themselves as asexual, then bisexual, then pansexual, then transgender all within a 6 month period).

- Yes
- No
- I don't know

If "yes", please describe the progression, noting the time frame in which it took place. For example, "during the course of 6 months" or "within two months", etc.

48. How would you rate your child's physical activity level?

- Sedentary
- Moderate
- Active

49. Does your child regularly engage in any of the following physical activities? (Check all that apply)

- Sports
- Dance
- Martial Arts (karate, tai kwon do, etc)
- Yoga
- Cardio exercise (walking, running, cardio machines, etc)
- Weight training
- None of the above
- Other (please specify)

50. On average, how many hours of sleep does your child get in a 24-hour period?

- Less than four hours
- 4-5 hours
- 6-7 hours
- 8-9 hours
- 10 or more hours
- I don't know

51. Does your child have any of the following obstacles to sleeping well at night? (Check all that apply)

- [ ] Has a job that requires him/her to work at night
- [ ] Socializes with other people at night
- [ ] Has difficulty falling asleep
- [ ] Has difficulty staying asleep
- [ ] Stays awake because he/she is on social media, the internet, or gaming
- [ ] None of the above
- [ ] I don't know.
- [ ] Other (please specify)

52. At what age did your child first have unsupervised access to the Internet?

If you would like to add more information, you may do so here.

53. At what age did your child first have access to their own smartphone?

If you would like to add more information, you may do so here.

54. In a typical day, approximately how many hours does your child spend using social media and the Internet?

If you would like to add more information, you may do so here.

55. What websites, blogs,etc do they use most frequently?

56. Are there particular websites, blogs, or bloggers, etc they follow/visit that are specific to transgender issues? What are they?

- ( ) Yes
- ( ) No
- ( ) I don't know

If "yes", please list the sites, blogs or bloggers.

57. Did you notice any changes in your child’s social media/Internet use in the period of time before they announced that they were transgender? (Choose the best answer)

- My child had a decrease in social media/Internet use in the period of time before they came out as transgender.
- My child had no change in his/her social media/Internet use in the period of time before they came out as transgender.
- My child had an increase in social media/Internet use in the period of time before they came out as transgender.
- I don't know.

If you would like to add more information, you may do so here.

58. Regarding Internet behavior, does your child? (Check all that apply)

- Lose track of time while online
- Sacrifice needed hours of sleep to spend time online
- Become agitated or angry when online time is interrupted
- Spend time online in place of homework or chores
- Prefer to spend time online rather than with friends or family
- Disobey time limits that have been set for internet usage
- Lie about amount of time spent online or “sneaks” online when no one is around
- Form new relationships with people he or she has met online (and does not know in person)
- Seem preoccupied with getting back online when away from the computer
- Lose interest in activities that were enjoyable before he or she had online access
- Become irritable, moody or depressed when not online
- Become irritable, moody or depressed the longer they are online
- None of the above
- Other (please specify)

59. Please check any of the following sources that you think were influential to your child's becoming gender dysphoric? (Check all that apply)

- [ ] Tumblr
- [ ] Reddit
- [ ] DeviantART
- [ ] Youtube transition videos
- [ ] Facebook
- [ ] cosplay communities
- [ ] a home-schooling community
- [ ] a school-based activity
- [ ] a Gay Straight Alliance (GSA) club
- [ ] a religious community
- [ ] a person that they met online
- [ ] a community/group of people that they met online
- [ ] a gaming community
- [ ] a person that they know in person (not online)
- [ ] a group of friends that they know in person
- [ ] a therapist
- [ ] a group therapy setting
- [ ] a family member
- [ ] None of the above
- [ ] Other, Please describe.

60. Has your child been formally identified as?

- ( ) Academically gifted
- ( ) Learning disabled
- ( ) Both
- ( ) Neither

61. Has there been a change in your child's grades/academic performance before, during, or after he/she came out as transgender? Please describe.

62. Did your child suffer a stressful or traumatic event shortly before becoming gender dysphoric? (Stressful event may include parents getting a divorce, moving to a new home, relationship issues, a break-up, an assault, etc)

- ○ Yes
- ○ No
- ○ I don't know
- ○ Other

Please specify

63. Has your child been diagnosed with any of the following BEFORE they became gender dysphoric? (Check all that apply)

- ☐ Depression
- ☐ Anxiety
- ☐ Obsessive Compulsive Disorder
- ☐ Bipolar Disorder
- ☐ Autism Spectrum Disorders (Autism, PDD-NOS, Asperger's)
- ☐ Attention Deficit Hyperactivity Disorder (ADHD/ADD)
- ☐ Eating Disorders
- ☐ Psychosis
- ☐ None of the above
- ☐ Other (please specify)

64. Has your child been diagnosed with any of the following DURING or AFTER they became gender dysphoric? (Check all that apply)

- ☐ Depression
- ☐ Anxiety
- ☐ Obsessive Compulsive Disorder
- ☐ Bipolar Disorder
- ☐ Autism Spectrum Disorders (Autism, PDD-NOS, Asperger's)
- ☐ Attention Deficit Hyperactivity Disorder (ADHD/ADD)
- ☐ Eating Disorders
- ☐ Psychosis
- ☐ None of the above
- ☐ Other (please specify)

65. If you checked any boxes in the TWO previous questions, please describe what age your child was diagnosed for each condition.

66. Is there a family history of mental illness? Please describe.

- Yes
- No
- I don't know

If "yes", please describe.

67. How would you rate your subjective sense of your child's mental well-being since they announced that they were transgender?

- My child's mental well-being has been much better since coming out as transgender.
- My child's mental well-being has been somewhat better since coming out as transgender.
- My child's mental well-being has been unchanged or mixed since coming out as transgender.
- My child's mental well-being has been somewhat worse since coming out as transgender.
- My child's mental well-being has been much worse since coming out as transgender.

If you would like to add more information, you may do so here.

68. How would you describe your relationship with your child since they announced that they were transgender?

- My relationship with my child is much better since they came out as transgender.
- My relationship with my child is somewhat better since they came out as transgender.
- My relationship with my child is unchanged or mixed since they came out as transgender.
- My relationship with my child is somewhat worse since they came out as transgender.
- My relationship with my child is much worse since they came out as transgender.

If you would like to add more information, you may do so here.

69. How would you describe your child's grades/academic performance since they announced that they were transgender?

- ◯ My child's grades/academics are much better since they came out as transgender.
- ◯ My child's grades/academics are somewhat better since they came out as transgender.
- ◯ My child's grades/academics are mixed or unchanged since they came out as transgender.
- ◯ My child's grades/academics are somewhat worse since they came out as transgender.
- ◯ My child's grades/academics are much worse since they came out as transgender.
- ◯ N/A

70. Since announcing that they were transgender, has your child...? (Check all that apply)

- ☐ Refused to speak to you
- ☐ Tried to run away
- ☐ Withdrawn from the family
- ☐ Told other people or posted on social media that you are "transphobic", "abusive", or "toxic" because you don't agree with them about their assessment that they are transgender
- ☐ Defended the practice of lying to or withholding information from therapists or doctors in order to obtain hormones for transition more quickly
- ☐ Dropped out of high school
- ☐ Needed to take a leave of absence from college or university
- ☐ Dropped out of college or university
- ☐ Been unable to obtain a job
- ☐ Been fired from a job
- ☐ Been unable to hold a job
- ☐ None of the above

If you would like to add additional information, you may do so here.

71. If you checked any choices in the previous question, is this behavior a significant change for your child from their baseline behavior?

- ◯ I did not check any choices for the previous question.
- ◯ Yes
- ◯ No

If you would like to add more information, you may do so here.

72. Has your child engaged in non-suicidal self injury (NSSI) such as cutting or burning? (Check all that apply)

- [ ] My child has never engaged in NSSI.
- [ ] My child was engaging in NSSI before becoming gender dysphoric.
- [ ] My child engaged in NSSI after they became gender dysphoric
- [ ] My child engaged in NSSI after they came out as transgender
- [ ] I don't know
- [ ] Other

If you would like to add more information, you may do so here.

73. Has you child called you “transphobic”, “bigoted”, (or another name to describe intolerance to transgender people) for any of the following reasons? (Check all that apply)

- [ ] Disagreeing with your child’s assessment that they are transgender
- [ ] Calling your child by his/her birth name
- [ ] Referring to your child by the pronouns (he/him or she/her) that they used to use before announcing they were transgender
- [ ] Telling your child that you think hormones and/or surgery would not be helpful to them
- [ ] Telling your child that you think hormones and/or surgery would be harmful to them
- [ ] Recommending therapy for basic mental health issues (not related to gender)
- [ ] Recommending a comprehensive evaluation before starting hormones and/or surgery
- [ ] Recommending that your child take more time to figure out if their feelings of gender dysphoria persist or go away
- [ ] Recommending that your child work on other mental health issues first to determine if they are they cause of their dysphoria
- [ ] Expressing concerns for your child’s future if they take hormones and/or have surgery
- [ ] Refusing to drive your child to a gender therapist, gender clinic or physician for the purpose of transitioning
- [ ] Refusing to pay for appointments, medications, surgery related to transition
- [ ] None of the above
- [ ] Other (please specify)

74. Has your child received advice from a person online (or people online) about the following topics? (Check all that apply)

- [ ] How to tell if they are transgender
- [ ] The reasons that they should transition right away
- [ ] That if they waited to transition they would regret it
- [ ] That if they didn't transition immediately they would never be happy
- [ ] That if their parents did not agree to take them for hormones, that the parents are 'abusive" and "transphobic"?
- [ ] That if their parents are reluctant to take them for hormones, that they should use the "suicide narrative" to convince them (telling the parents that there is a high rate of suicide in transgender teens)
- [ ] That it is acceptable to lie to or withhold information about one's medical or psychiatric history from a doctor or therapist in order to get hormones/get hormones faster
- [ ] What to say and what NOT to say to a doctor or therapist in order to convince them to provide hormones
- [ ] How to get money from others online in order to pay for medications, etc
- [ ] How to get hormones from online sources
- [ ] How to hide hormones from parents
- [ ] How to order physical items (binders, packers, etc) without parents finding out
- [ ] How to hide physical items (binders, packers, etc) from parents
- [ ] How to hide or make excuses for physical changes
- [ ] Medical advice about the risks and benefits of hormones
- [ ] Medical advice about the risks and benefits of surgery
- [ ] I don't know if my child received advice from someone online about these topics

If you would like to add additional information, you may do so here.

75. How did your child first tell you that they were transgender?

- ( ) Speaking to you in person
- ( ) Speaking to you over the telephone
- ( ) Speaking to you via Skype or FaceTime
- ( ) In a letter
- ( ) In an email
- ( ) In a text message
- ( ) I heard from someone else.
- ( ) Other (please specify)

76. When your child first told you that they were transgender, did you suspect that they used language that they found online? If so, please describe.

- Yes
- No
- N/A

Please describe. If you could share the language used (without using your child's name) that would be helpful.

77. Did your child ? (Check all that apply)

- Tell you that they wanted to see a gender therapist or go to a gender clinic
- Go on their own to visit a gender therapist or gender clinic
- Tell you that they wanted to take hormones for transition purposes
- Obtain hormones on their own for transition purposes
- Tell you that they wanted surgery for transition purposes
- Have any surgeries for transition purposes
- Tell you about the issue of suicide in transgender teens when asking for appointments, hormones or surgery
- Bring up the issue of suicide in transgender teens as a reason that you should agree to appointments, hormones or surgery
- None of the above

If you would like to add more information, you may do so here.

78. How soon after your child announced that they were transgender did they tell you they wanted to transition (with hormones or surgery)?

- Not applicable
- At the same time that that announced they were transgender
- Less than a week after announcing they were transgender
- Between one week and one month after announcing they were transgender
- About 2 months after announcing they were transgender
- About 3 months after announcing they were transgender
- About 4 months after announcing they were transgender
- About 5 months after announcing they were transgender
- About 6 months after announcing they were transgender
- More than 6 months after announcing they were transgender
- Other (please specify)

79. Has your child seen a gender therapist, gone to a gender clinic, or seen a physician for the purpose of beginning transition?

- ○ Yes
- ○ No
- ○ I don't know

If you would like to add more information, you may do so here.

80. Was your child willing to work on basic mental health issues with a general psychologist or therapist FIRST, before going to a gender therapist, gender clinic, or a physician for the purpose of transitioning?

- ○ Yes
- ○ No
- ○ I don't know
- ○ N/A

81. If your child has seen a gender therapist, gone to a gender clinic, or seen a physician for the purpose of beginning transition, were you allowed to attend the meeting, provide information about your child, and have any concerns you might have had addressed? (Check all that apply)

- ☐ My child has not seen a gender therapist, gone to a gender clinic, or seen a physician for the purposes of beginning transition.
- ☐ I was able to attend the appointment with my child.
- ☐ I was not able to attend the appointment with my child.
- ☐ I was not permitted to attend the appointment with my child.
- ☐ I was not informed of the appointment.
- ☐ I was able to speak with the therapist, physician or clinic staff.
- ☐ I was not able to speak with the therapist, physician or clinic staff.
- ☐ I was able to provide additional information about my child to the therapist, physician, or clinic staff.
- ☐ I was not able to provide additional information about my child to the therapist, physician, or clinic staff.
- ☐ If I had concerns about my child's diagnosis or course of treatment, my perspective was understood and taken into account as valid.
- ☐ The therapist, physician, or clinic staff addressed any concerns I might have had.
- ☐ The therapist, physician, or clinic staff did not address concerns I might have had.
- ☐ Other

If you would like to add more information, you may do so here.

82. If your child went to a gender therapist, a gender clinic, or saw a physician for the purpose of beginning transition, in your opinion, did the therapist/physician/clinic staff explore issues of mental health, previous trauma or any alternative causes for dysphoria before proceeding?

- ◯ My child did not go to a gender therapist, a gender clinic, or see a physician for the purpose of transitioning.
- ◯ The therapist/physician/clinic staff explored issues of mental health, previous trauma or any alternative causes for dysphoria before proceeding.
- ◯ The therapist/physician/clinic staff did NOT explore issues of mental health, previous trauma or any alternative causes for dysphoria before proceeding.
- ◯ I don't know.

If you would like to add more information, you may do so here.

83. If your child went to a gender therapist, gender clinic, or a physician for the purposes of transitioning, did the therapist, physician, or clinic staff ask for any of your child's medical records (such as pediatric records or psychologist records) before proceeding with treatment?

- ◯ My child did not go to a gender therapist, gender clinic, or physician for the purposes of transitioning.
- ◯ Yes
- ◯ No
- ◯ I don't know

84. If your child went to a gender therapist, gender clinic, or a physician for the purposes of transitioning, did your child receive a prescription for cross sex hormones and/or puberty blockers at their first visit?

- ◯ My child did not go to a gender therapist, gender clinic, or physician for the purposes of transitioning.
- ◯ Yes
- ◯ No
- ◯ I don't know

If your child received prescriptions for cross sex hormones or puberty blockers at a visit other than the first, please describe approximately how many visits before they received their prescription(s).

85. If your child went to a gender therapist, gender clinic, or a physician for the purposes of transitioning, do you suspect that your child misrepresented their history to the therapist, physician or staff? (For example, did they tell a story of their childhood that was very different than what occurred? Did they omit important information about mental illness or trauma?)

- ◯ My child did not go to a gender therapist, gender clinic or a physician for the purposes of transition.
- ◯ I am positive that my child relayed their full history accurately and completely.
- ◯ I am reasonably sure that my child relayed their full history accurately and completely.
- ◯ I am reasonably sure that my child misrepresented or omitted parts of their history.
- ◯ I am positive that my child misrepresented or omitted parts of their history.
- ◯ I don't know
- ◯ Other

If you would like to add more information, you may do so here.

86. Has your child transitioned in any way? (Check all that apply)

- ☐ My child has changed his/her hair.
- ☐ My child is using different pronouns.
- ☐ My child has asked people to call him/her by a different name.
- ☐ My child dresses in a different style of clothes more traditionally worn by the other gender.
- ☐ My child has legally changed their name on government documents.
- ☐ My child has taken puberty blockers.
- ☐ My child has taken cross sex hormones.
- ☐ My child has taken anti-androgen medication.
- ☐ My child has had surgery for transition purposes.
- ☐ none of the above.

If you would like to add more information, you may do so here.

87. Is your child still identifying as transgender?

- ◯ Yes
- ◯ No
- ◯ I don't know.
- ◯ N/A

88. If the answer to the previous question is yes, how long has your child been identifying as transgender? Please describe.

89. If your child identified as transgender and is not identifying as transgender now, how long did they identify? How long has it been since they stopped identifying? Please describe.

90. If you would like to, please tell us more about any aspect of your family's experience or anything you think might be helpful for our research.

Thank you for your participation in this study.
